# Identification and validation of single-sample breast cancer radiosensitivity gene expression predictors

**DOI:** 10.1186/s13058-018-0978-y

**Published:** 2018-07-04

**Authors:** Martin Sjöström, Johan Staaf, Patrik Edén, Fredrik Wärnberg, Jonas Bergh, Per Malmström, Mårten Fernö, Emma Niméus, Irma Fredriksson

**Affiliations:** 1 Faculty of Medicine, Department of Clinical Sciences Lund, Oncology and Pathology, Lund University, Lund, Sweden; 2 Department of Haematology, Oncology and Radiation Physics ,Skåne University Hospital, Lund, Sweden; 3 Department of Theoretical Physics and Computational Biology, Lund University, Lund, Sweden; 4 Department of Surgical Sciences, Uppsala University, Uppsala, Sweden; 5 Department of Oncology and Pathology, Cancer Center Karolinska, Karolinska Institutet, Stockholm, Sweden; 6 Department of Oncology, Karolinska University Hospital, Radiumhemmet, Stockholm, Sweden; 7 Faculty of Medicine, Department of Clinical Sciences Lund, Surgery, Lund University, Lund, Sweden; 8 Department of Surgery, Skåne University Hospital, Lund, Sweden; 9 Department of Molecular Medicine and Surgery, Karolinska Institutet, Stockholm, Sweden; 10 Department of Breast- and Endocrine Surgery, Karolinska University Hospital, Stockholm, Sweden

**Keywords:** Breast cancer, Gene expression, Radiotherapy, Radiosensitivity, Radioresistance, Ipsilateral breast tumor recurrence, Local recurrence, Nanostring, nCounter

## Abstract

**Background:**

Adjuvant radiotherapy is the standard of care after breast-conserving surgery for primary breast cancer, despite a majority of patients being over- or under-treated. In contrast to adjuvant endocrine therapy and chemotherapy, no diagnostic tests are in clinical use that can stratify patients for adjuvant radiotherapy. This study presents the development and validation of a targeted gene expression assay to predict the risk of ipsilateral breast tumor recurrence and response to adjuvant radiotherapy after breast-conserving surgery in primary breast cancer.

**Methods:**

Fresh-frozen primary tumors from 336 patients radically (clear margins) operated on with breast-conserving surgery with or without radiotherapy were collected. Patients were split into a discovery cohort (*N* = 172) and a validation cohort (*N* = 164). Genes predicting ipsilateral breast tumor recurrence in an Illumina HT12 v4 whole transcriptome analysis were combined with genes identified in the literature (248 genes in total) to develop a targeted radiosensitivity assay on the Nanostring nCounter platform. Single-sample predictors for ipsilateral breast tumor recurrence based on a k-top scoring pairs algorithm were trained, stratified for estrogen receptor (ER) status and radiotherapy. Two previously published profiles, the radiosensitivity signature of Speers et al., and the 10-gene signature of Eschrich et al., were also included in the targeted panel.

**Results:**

Derived single-sample predictors were prognostic for ipsilateral breast tumor recurrence in radiotherapy-treated ER+ patients (AUC 0.67, *p* = 0.01), ER+ patients without radiotherapy (AUC = 0.89, *p* = 0.02), and radiotherapy-treated ER- patients (AUC = 0.78, *p* < 0.001). Among ER+ patients, radiotherapy had an excellent effect on tumors classified as radiosensitive (*p* < 0.001), while radiotherapy had no effect on tumors classified as radioresistant (*p* = 0.36) and there was a high risk of ipsilateral breast tumor recurrence (55% at 10 years). Our single-sample predictors developed in ER+ tumors and the radiosensitivity signature correlated with proliferation, while single-sample predictors developed in ER- tumors correlated with immune response. The 10-gene signature negatively correlated with both proliferation and immune response.

**Conclusions:**

Our targeted single-sample predictors were prognostic for ipsilateral breast tumor recurrence and have the potential to stratify patients for adjuvant radiotherapy. The correlation of models with biology may explain the different performance in subgroups of breast cancer.

**Electronic supplementary material:**

The online version of this article (10.1186/s13058-018-0978-y) contains supplementary material, which is available to authorized users.

## Background

Precision medicine has been the focus of breast cancer research during recent decades. As breast cancers are detected at an earlier stage, and treatment has improved, the emphasis to avoid over treatment in addition to under-treatment has increased [1]. Currently, the majority of primary breast cancers are treated with breast-conserving surgery (BCS), and the patient is generally offered adjuvant treatment. Prognostic and treatment-predictive biomarkers based on traditional immunohistochemical analysis (IHC), or more modern molecular techniques such as gene expression profiling, are presently used to guide the use of adjuvant endocrine therapy, chemotherapy and anti-human epidermal growth factor receptor 2 (HER2)-directed therapy [2]. However, there is no diagnostic procedure to guide treatment with adjuvant radiotherapy (RT) after BCS, which is administered to a majority of patients. This is despite the knowledge that most patients who undergo BCS will remain recurrence-free without RT for at least 10 years, and around 20% will suffer a recurrence within 10 years despite RT [3]. Traditional clinicopathologic variables and IHC markers have been unable to identify patients that could be spared RT [3–5], although studies are ongoing to find patients with risk of recurrence low enough to avoid RT (e.g. the LUMINA study, NCT02653755, and the PRIMETIME study [6]).

Several attempts have been made to create gene expression-based classifiers to predict response to RT after BCS, or to estimate the risk of recurrence with or without RT [7–11]. Most recently, Speers et al. presented the radiosensitivity signature (RSS), a 51-gene random forest model to classify tumors as radioresistant or radiosensitive [12]. Tramm et al. presented a 4-gene classifier predicting the response to RT after mastectomy [13]. Torres-Roca et al. presented the radiosensitivity index (RSI), a linear model based on the rank of genes in individual samples, which has been validated in several cancer types, including breast cancer [8]. The same authors have also advanced the model by combining RSI with the linear-quadratic model for the genomic-adjusted radiation dose (GARD) [14]. In addition, genome instability is considered to sensitize cancer cells to treatment in general, and a centromere and kinetochore gene expression score was suggested to predict response to RT [15]. Taken together, promising results have been presented, but no profile or marker is yet in clinical use.

There are several reasons why gene expression profiles have not been introduced in clinical routine. First, the clinical value and cost-effectiveness has not been proven, as reported profiles lack extensive independent validation, and to date, no prospective trial or studies from existing randomized clinical trials have been presented, except in the mastectomy setting [13]. Second, few of the current profiles have been tested on technical platforms able to handle samples with low-quality RNA, such as RNA extracted from formalin-fixed paraffin-embedded (FFPE) tissue, which would greatly improve the clinical utility. Third, it has been hard to validate profiles across platforms, although attempts have been made by e.g. scaling (RSS) or rank-based models (RSI). Finally, breast cancer is a heterogeneous disease, and the response to RT and the pathways associated with radioresistance may be different in different subgroups. Indeed, this was shown when Torres-Roca et al. presented the follow-up study of RSI in estrogen receptor positive (ER+) and estrogen receptor negative (ER-) breast cancer, and only could validate previous findings in ER- tumors [16]. Interestingly, RSI was recently further shown to correlate with immune response genes, which may partly explain the subgroup-specific performance, as the immune response is more important for prognosis in ER- breast cancer [17, 18].

In this study, we aimed to address these issues and created a targeted radiosensitivity gene expression assay using the Nanostring nCounter platform, which is suitable for low quality RNA samples. Based on the targeted assay, we created single-sample predictors (SSPs) using a k-top scoring pairs (k-TSP) algorithm [19]. The SSPs were validated to be prognostic for ipsilateral breast tumor recurrence (IBTR) in samples of low RNA quality from a study cohort, and further validated in public data. The SSPs also showed potential to stratify patients for RT. In addition, the panel included the genes described for RSS and a surrogate score for RSI (referred to as the 10-gene signature, 10-GS). The previously reported signatures were prognostic for IBTR, and partially predictive of RT, but their performance was dependent on ER status. Finally, we showed that the biology behind the different models and predictors may explain this difference.

## Methods

### Patients and samples

Patients with invasive breast cancer radically operated on (clear margins) with BCS in three of six healthcare regions in Sweden (South, Uppsala-Örebro and Stockholm) between 1983 and 2009, and with fresh-frozen tissue available, were included (*N* = 336). Patients were excluded if they had multifocal cancer (defined as > 20 mm between tumors), neoadjuvant treatment or prior malignancy (excluding basal-cell carcinoma of the skin, in-situ cervical cancer and other curatively treated cancer at least 5 years prior to the breast cancer). First, all patients with a later IBTR were selected as cases (*N* = 144). Next, controls were selected as patients without any recurrence for at least the same time as the time to IBTR for the matched case, and were matched for RT and ER status (*N* = 192). Median follow-up time was 13.1 years in patients without IBTR (controls), and median time to IBTR was 4.4 years in patients with IBTR (cases). Systemic adjuvant treatment was not part of the inclusion criteria and was administered according to regional treatment programs at the time. The study was approved by the Ethics committee of Lund University (2010-127).

### RNA extraction

**Fig. 1:**
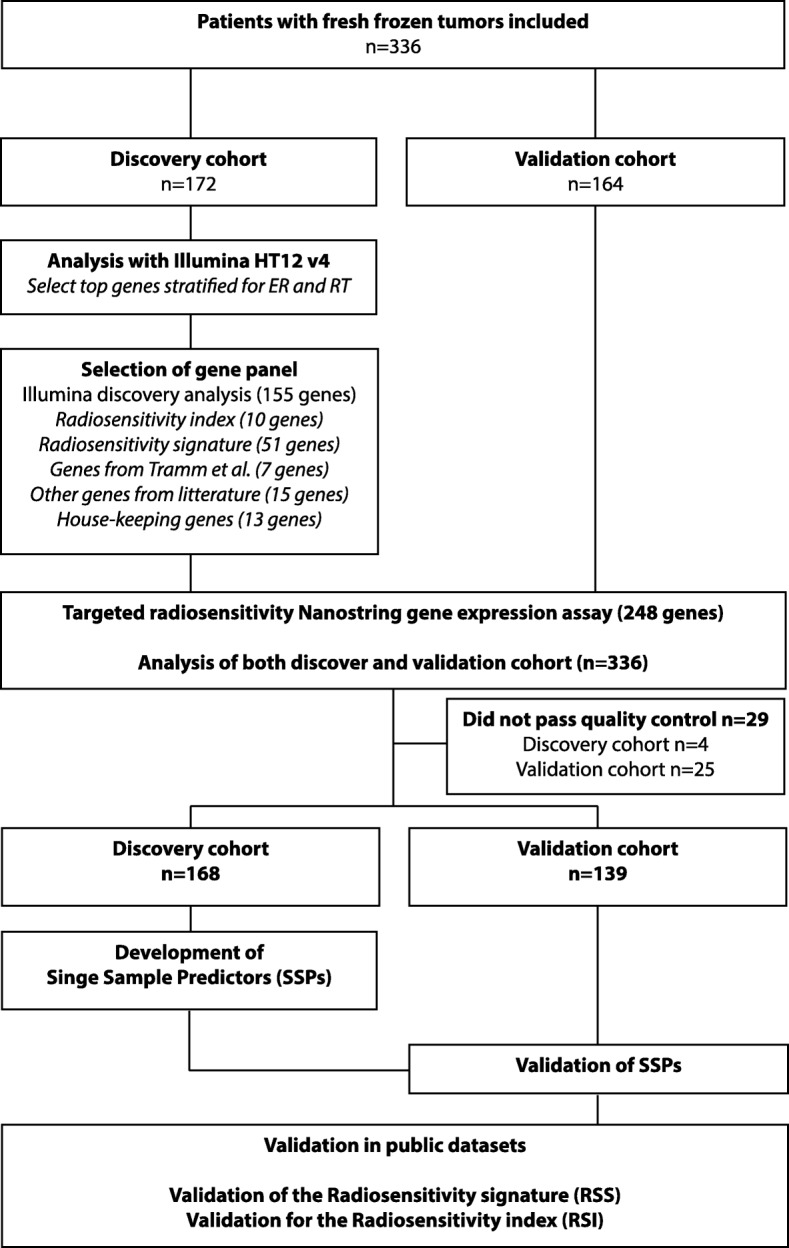
Project overview. Samples were split into a discovery cohort and a validation cohort. The discovery cohort was analyzed with the Illumina HT12 v4 whole transcriptome microarray. Top discriminating genes for ipsilateral breast tumor recurrence were combined with genes from the literature and previous signatures for a radiosensitivity gene panel. A targeted assay was developed, and both the discovery cohort and the validation cohort were analyzed. Single-sample predictors were developed in the discovery cohort and validated in the validation cohort and in public datasets. Finally, the previously published signatures were tested in all our data. ER, estrogen receptor; RT, adjuvant radiotherapy

RNA was extracted from approximately 30 mg of fresh-frozen tissue using commercially available extraction kits, either the Qiagen AllPrep kit, or the Qiagen RNEasy lipid tissue kit, according to the manufacturer’s instructions (Qiagen, Hilden, Germany). Cancer content was confirmed microscopically and samples without cancer cells were excluded. Integrity and amount of RNA was measured; samples from one of the three biobank centers had RNA of lower quality, which most likely can be explained by degradation during the transportation process (Additional file 1: Figure S3). We chose to use the higher-quality samples from two centers as a discovery cohort (*N* = 172), and the lower-quality samples from one center as a validation cohort (*N* = 164) (Fig. 1 and Table 1). For more details, see Additional file 2.

**Table 1:** Patient and tumor characteristics

	Discovery cohort	Validation cohort
Total number of patients	172	164
Analyzed with Illumina HT12 v4	172	0
Analyzed with targeted nCounter panel	172	164
Included in the final analysis	168	139
Radically operated on (clear margins)		
Yes	168 (100%)	139 (100%)
No	0	0
Extensive intraductal component (EIC)		
Yes	9	7
No	109	90
Missing	50	42
Ipsilateral breast tumor recurrence (IBTR)		
Yes	68	62
No	100	77
Tumor size mm, median (min-max)	18 (3-45)	17 (3-35)
Lymph node status		
Node negative	125 (78%)	108 (78%)
Node positive	35 (22%)	29 (22%)
Missing	8	2
Estrogen receptor (ER) status		
Positive	119 (71%)	118 (85%)
Negative	49 (29%)	21 (15%)
Histological grade		
1	16 (17%)	12 (19%)
2	46 (50%)	24 (39%)
3	30 (33%)	26 (42%)
Missing	76	77
Subtype		
Luminal A	70 (42%)	60 (43%)
Luminal B	42 (25%)	29 (21%)
Basal-like	37 (22%)	12 (9%)
Human epidermal growth factor receptor 2 (HER2)-enriched	19 (11%)	38 (27%)
Radiotherapy		
Yes	116 (69%)	119 (86%)
No	52 (31%)	20 (14%)
Chemotherapy		
Yes	34 (20%)	31 (23%)
No	133 (80%)	105 (77%)
missing	1	3
Endocrine therapy		
Yes	60 (35%)	91 (65%)
No	108 (65%)	46 (35%)
missing	0	1
Follow-up time		
Median time (range) to IBTR in cases, years	3.7 (0.7-18.7)	4.4 (0.1-22.5)
Median follow-up time (range) in controls, years	13.2 (3.0-19.6)	12.6 (1.7-26.0)

### Gene expression analysis in the discovery cohort

The discovery cohort (*N* = 172) was analyzed using Illumina HT12 v4 microarrays (Illumina, San Diego, CA, USA). The input amount was 575 ng of total RNA and RNA was hybridized on three plates. Samples were processed in a randomized order and the data have been deposited in Gene Expression Omnibus (GEO) [GEO:GSE103746].

### Data analysis in the discovery cohort

All data analyses were performed using R [20] (explicitly outlined in Additional file 2). Briefly, the Illumina HT12 v4 array data was normal-exponential background corrected, quantile-normalized and log2-transformed with an offset of 16 added to avoid negative values using the limma package [21], as previously suggested [22]. The data were batch-effect corrected for hybridization plate and biobank center using the sva package [23]. Probes were filtered based on quality and a variance filter was applied to limit the number of probes to 5000. Tumors were stratified for ER and RT status creating four groups (ER+RT+, ER+RT-, ER-RT+, ER-RT-). A random forest model with double-loop cross-validation and recursive feature elimination based on the caret R package [24] was used to rank the importance of candidate genes, and select the number of genes to analyze further.

### Creation of a targeted radiosensitivity gene panel

Genes included in the targeted panel were selected based on the discriminating performance of cases versus controls in the discovery cohort (*N* = 155). We further added the genes included in the previously published signatures RSI (*N* = 10), RSS (*N* = 51) and the genes described by Tramm et al. (*N* = 7) [8, 12, 13]. We also added genes associated with risk of IBTR, radioresistance or breast cancer biology identified in the literature, e.g. hormone and growth factor receptors (*ESR1*, *PGR*), human epidermal growth factor receptor 2 (*ERBB2*), proliferation genes (*MKI67* and *AURKA*), and genes related to hypoxia, apoptosis and DNA repair (*N* = 15) [25–30]. Housekeeping genes were added for purposes of normalization (*N* = 13). In total, 248 genes were selected for the targeted gene expression panel (Fig. 1). For details see Additional file 2, and Additional file 3: Table S1.

### Gene expression analysis with the targeted radiosensitivity panel

Both the discovery cohort (*N* = 172) and validation cohort (*N* = 164) were analyzed in a randomized order with a custom-designed Nanostring nCounter panel (Nanostring Technologies, Seattle, WA, USA). The Nanostring probes were created with standard chemistry XT-formulation and designed and produced by the manufacturer (Nanostring). Analysis-ready probes were analyzed using the Prepstation and Digital analyzer (Nanostring), according to the manufacturer’s instructions. Gene expression data have been deposited to GEO [GEO:GSE10374]. For more details, see Additional file 2.

### Public datasets

Two public datasets were analyzed [11, 31]. The dataset of Servant et al. was based on anlysis using the Illumina HT12 v3 in a cohort of 343 patients who underwent BCS and were treated with RT. The dataset of van de Vijver et al. included 295 patients who underwent either BCS or modified radical mastectomy. RT was given when indicated, and gene expression was analyzed by a 25,000-gene oligonucleotide dual-channel array.

### Data analysis in the targeted radiosensitivity panel

The data were quality-filtered resulting in 7 probes and 29 samples removed (4 from the discovery cohort and 25 from the validation cohort) and normalized for positive control probes and housekeeping genes (Fig. 1). SSPs to classify samples as high risk or low risk of IBTR were trained in the discovery cohort in each of the four groups (ER + RT+, ER + RT-, ER-RT+ and ER-RT-) using the switchbox R package [19]. The SSPs were based on a k-TSP algorithm that compares the relative expression of genes within a sample and creates rules in the form gene A > gene B. The default settings of the switchbox package were used, which selects the optimal number of gene pairs by cross-validation in the discovery cohort, [32] and uses the majority vote as cut point without any weighting of the pairs. The model was allowed to use all genes in the panel and minimum number of pairs to try for training was set to 100 pairs, as gene expression profiles have been shown to be more robust using higher number of genes [33]. This means that at least 200 genes were included in each SSP, and thus a combination of previously published genes and novel genes from our discovery analysis. The full set of genes and pairwise combination is presented in Additional file 4: Table S3. The locked models were then tested in the validation cohort and Kaplan-Meier curves, Cox regression models, and log-rank *p*-values were calculated using the survival R package [34], and receiver operating characteristics (ROC) analysis was performed using the pROC R package [35]. Endpoint was IBTR. RSS and a surrogate score for RSI (referred to as 10-GS) were calculated as described in the original publications [8, 12]. Proliferation scores were calculated as the geometric mean of expression values for *MKI67* and *AURKA*. Immune scores were calculated as the geometric mean of genes annotated as part of the immune response (*IRF1, IGKC, STAT1, OSMR, CCL19, RelA, IRF8, FGR, TNFRSF1B, C3*) in the online gene ontology tool PANTHER [36]. Correlation between the raw scores for the different models, and correlation with proliferation and immune scores were tested with Pearson correlation and linear modeling, with p-values calculated with a test for zero slope. For more details, see Additional files.

## Results

### Selection of genes and creation of a targeted radiosensitivity assay

The Illumina HT12 v4 microarray whole transcriptome gene expression data from the discovery cohort was analyzed stratified for ER status and RT, creating four groups (ER + RT+, ER + RT-, ER-RT+, ER-RT-). ROC analysis showed that optimal performance of the random forest models was achieved after including around 50 genes per model, with the AUC ranging from 0.67 to 0.85 depending on group, except for the ER-RT+ subgroup, where no signal was found (Additional file 5: Figure S1A and B). Based on their importance in these models, we selected 155 genes for further development of a targeted assay. To investigate the biology represented by the selected genes, hierarchical clustering was performed and correlated with known gene clusters (Additional file 2 and Additional file 6: Figure S2). Genes selected in the ER+ groups included genes correlated with proliferation, and genes selected in the ER- groups included genes correlated with immune response. However, for some clusters no correlation was found, and the genes may thus represent biological pathways more specific for radiosensitivity.

We added genes from three previously described radioresistance gene expression profiles in breast cancer to the 155 genes selected in the discovery analysis: these were the 10 genes forming the RSI, the 51 genes included in the RSS, and the 7 genes described by Tramm et al. [8, 12, 13]. We further added genes identified in the literature (Additional file 3: Table S1). Among these were genes associated with apoptosis (*BCL2*) [25], DNA-repair (*BRCA1*, *BRCA2* and survivin/*BIRC5*) [26, 27], the MET-HGF pair [28], hypoxia (*HIF1* and *HIF2*) [29] and *WRAP53* [30]. We also added genes important for breast cancer biology or subtyping (*ER, PGR, ERBB2, MKI67, AURKA and FOXC1*). Finally, we added 13 housekeeping genes previously used by Nanostring in their targeted gene expression assays (Additional file 3: Table S1). In total, 248 genes were selected for the development of a targeted assay.

### Training and validation of single-sample predictors with the targeted assay

**Fig. 2:**
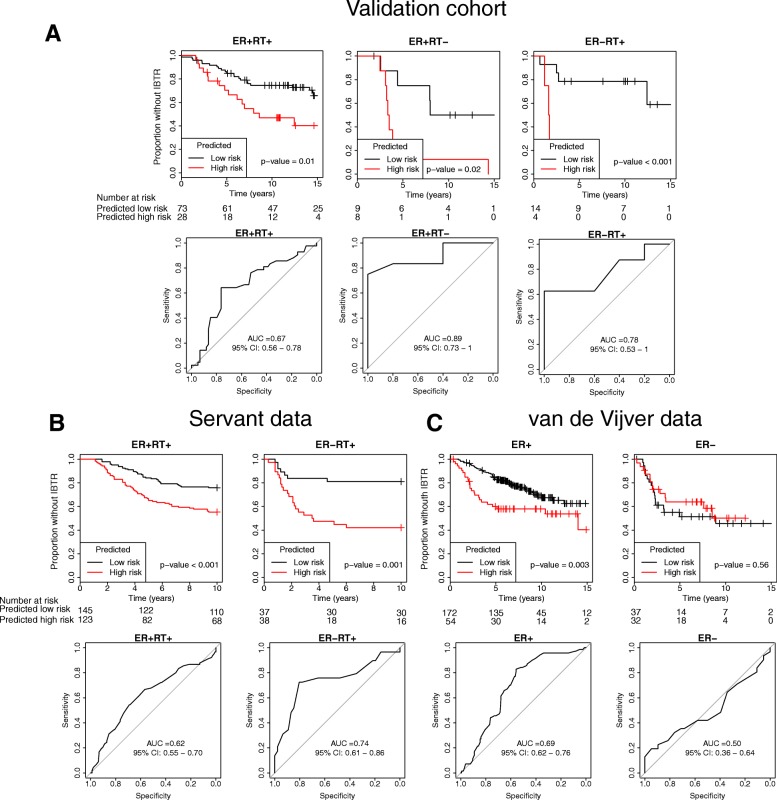
Validation of single-sample predictors (SSPs) in our validation cohort (**a**) and two publicly available datasets (**b** and **c**). The analysis was performed with data stratified for estrogen receptor (ER) status and adjuvant radiotherapy (RT). The endpoint was ipsilateral breast tumor recurrence (IBTR) and the SSPs were evaluated by survival analysis using the Kaplan-Meier method and log-rank test, and receiver operating characteristics (ROC) analysis with area under the curve (AUC) as a measurement of performance

Both the discovery cohort and the validation cohort were analyzed with the targeted Nanostring assay. SSPs were trained in the discovery cohort separately for the four groups created when stratifying for ER status and RT status (ER + RT+, ER + RT-, ER-RT+ and ER-RT-). The locked models were then applied in the validation cohort. The validation AUC was 0.67 for the SSP in ER + RT+ samples, 0.89 for the SSP in ER + RT- samples, and 0.78 for SSP in ER-RT+ samples. The ER-RT- group could not be analyzed due to too few samples (*N* = 3). The SSPs were significantly associated with IBTR in survival analysis (log-rank *p* = 0.01, *p* = 0.02 and *p* < 0.001, respectively) (Fig. 2a). Next, we tested the SSPs in two public datasets and mapped the genes to the respective platforms. Three genes were missing in the Servant et al. dataset, and 34 genes were missing in the van de Vijver dataset, and thus we used the SSPs without these gene pairs. All patients in the Servant et al. dataset were treated with RT and we could thus only test the ER + RT+ and ER-RT+ SSPs. Both SSPs were significantly predictive of IBTR (log-rank *p* < 0.001 and *p* = 0.001, respectively) with corresponding AUC values of 0.62 and 0.74 (Fig. 2b). The van de Vijver dataset also included a majority of RT-treated patients, and we therefore again tested the ER + RT+ and ER-RT+ SSPs. The ER + RT+ SSP was significantly predictive of IBTR (*p* = 0.003, AUC 0.69) but not the ER-RT+ SSP (*p* = 0.56, AUC 0.50) (Fig. 2c).

### Potential clinical application

**Fig. 3:**
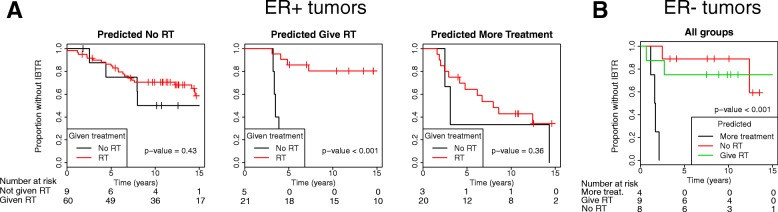
Application of single-sample predictors (SSPs) to stratify patients for treatment. The analysis was performed with data stratified for estrogen receptor (ER) status. SSPs developed in radiotherapy (RT)-untreated patients (RT-) were used to estimate the risk without giving RT. If they were predicted as having low risk of IBTR without RT, they were assigned to the “No-RT” groups. If predicted as high risk without RT, a SSP developed in RT+ tumors was applied. If predicted as having low risk with RT, they were assigned to the group “Give RT” and if predicted as having high risk with RT, they were assigned to the “More-treatment” group. The difference in risk of ipsilateral breast tumor recurrence with or without RT was visualized using the Kaplan-Meier method and tested with the log-rank test for ER+ tumors (**a**) and ER- tumors (**b**). Among ER- tumors (**b**), only two were RT- and we thus analyzed the prognostic effect of the groups assigned

The first set of analyses focused on prognostic predictors, either in RT+ patients where our SSPs may be regarded as radioresistance classifiers, or in RT- patients, in which the SSPs may be seen as a method for finding patients without the need for RT. However, the aim was to derive a classifier that can stratify patients into three groups: (1) those that could be spared RT, (2) those that benefit from and should be given RT and (3) those that are intrinsically radioresistant, and where other treatments strategies should be considered besides RT, e.g. mastectomy or more aggressive adjuvant systemic treatment. One strategy to stratify patients into the three treatment groups could be to apply our SSPs consecutively, such that we first determine which patients should be spared RT with a SSP developed in RT- patients. Patients predicted to have low risk of IBTR would be in the “No-RT” group. For the patients predicted as high risk of IBTR, the SSP developed in RT+ patients, and thus potentially testing radioresistance, could next be applied. Patients predicted as having low risk of IBTR when given RT would be in the “Give-RT” group, while patients predicted as having high risk of IBTR even with RT would be in the “More-treatment” group. To test this conceptual idea, we applied our SSPs consecutively in our validation cohort separately for ER+ and ER- tumors. For ER+ tumors, the No-RT group had no benefit from RT (*p* = 0.43), but did not have a low risk of developing IBTR (25% at 10 years) (Fig. 3a). The effect of RT was excellent in the Give-RT group (*p* < 0.001), while RT had no effect in the More-treatment group (*p* = 0.36), and the group had a substantially higher risk of IBTR than the No-RT group (55% at 10 years) (Fig. 3a). In a Cox model of the ER+ tumors including the variable of “Give RT vs No RT” and “Give more treatment”, RT and the interaction term between the prediction and RT, the interaction term was significant (HR_interaction_ = 0.12 95% CI 0.03–0.54, P_interaction_ = 0.001), further strengthening the treatment predictive potential (Additional file 7: Table S4). Among patients with ER- tumors, only two were RT-untreated, and we could thus only investigate the prognostic effect in this group. Those that were predicted as More treatment had a significantly higher rate of IBTR than the patients in the No-RT and Give-RT groups (*p* < 0.001) (Fig. 3b).

### Analysis of previously published profiles in our data

**Fig. 4:**
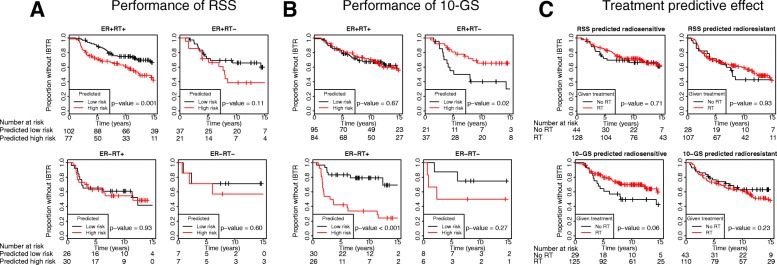
Performance of the radiosensitivity signature (RSS) (**a**) and the 10-gene score (10-GS) (**b**) in the Nanostring data generated with the targeted radiosensitivity gene expression assay. Tumors classified as a case by RSS, or above the median 10-GS score, were regarded high risk. The prognostic performance was evaluated with the Kaplan-Meier method and log-rank test for endpoint ipsilateral breast tumor recurrence, stratified for estrogen receptor (ER) status and radiotherapy (RT). The treatment predictive effect was evaluated by analyzing the effect of RT in samples classified as radioresistant or radiosensitive by the respective classifiers (**c**)

The RSS described by Speers et al. was applied to our entire dataset created with the targeted assay (*N* = 307), as described in the original publication. There was an overall association with IBTR in the full dataset (log-rank *p* = 0.001, AUC of 0.59). When it was applied as stratified for ER and RT, it remained significant only in the ER + RT+ group (*p* = 0.001, AUC 0.58) (Fig. 4a). The 10-GS, based on the genes included in the RSI, was applied to the targeted dataset as described in the original publications, with the change that the cut point was set to the median value, as we have enriched for patients with later IBTR in this dataset. Overall it did not predict the development of IBTR (log-rank *p* = 0.20, AUC 0.51). However, stratified for ER and RT, it performed well in the ER-RT+ group (log-rank *p* < 0.001, AUC 0.70) (Fig. 4b). Further, high risk/radioresistance, as predicted by 10-GS, was significantly associated with fewer instances of IBTR in the ER + RT- group (log-rank *p* = 0.02, AUC 0.70 when changing the direction of analysis) (Fig. 4b).

We also tested the treatment predictive effect of RSS and 10-GS, i.e. the effect of RT in those predicted to be radioresistant or radiosensitive, respectively. Neither of the RSS groups had an effect of RT (*p* = 0.71 and *p* = 0.93, respectively) (Fig. 4c). For the 10-GS, on the other hand, RT had no effect on the samples predicted to be radioresistant (*p* = 0.23), while there was an effect of RT in the samples predicted to be radiosensitive (*p* = 0.06) (Fig. 4c). A Cox regression model including RT, 10-GS and the interaction term between RT and 10-GS showed that the interaction term was significantly predictive of IBTR (p_interaction_ = 0.03), suggesting a treatment predictive effect of the 10-GS.

### Comparison of models and association with underlying biology

**Fig. 5:**
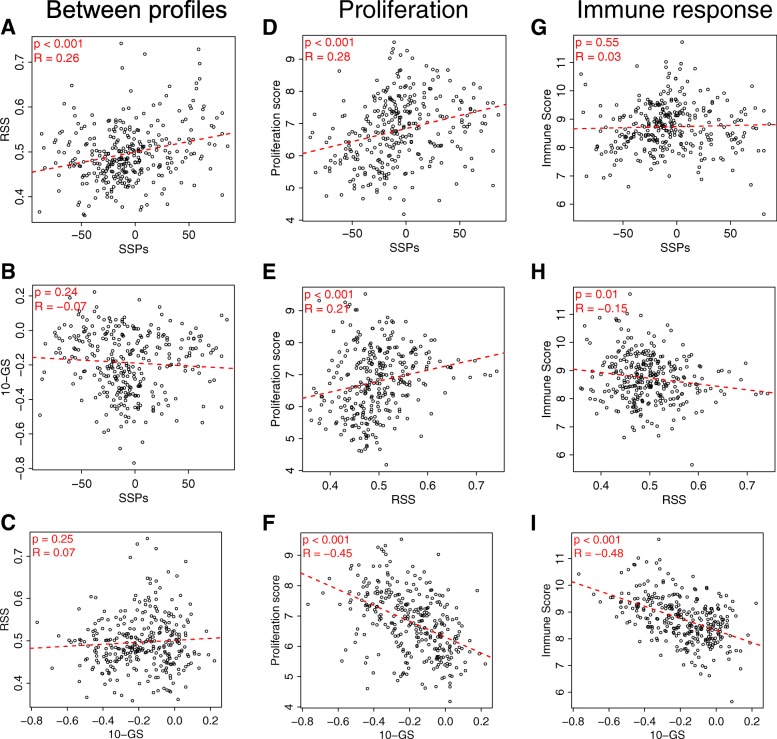
Correlation between our single-sample predictors (SSPs), the radiosensitivity signature (RSS) and the 10-gene signature (10-GS) in the combined discovery and validation data from the targeted radiosensitivity assay (**a**-**c**). The samples are classified with the corresponding SSPs, i.e. stratified for estrogen receptor and radiotherapy status. The different profiles were further correlated with a proliferation score calculated as the geometric mean of the expression of *AURKA* and *MKI67* (**d**-**f**), and with an immune score calculated as the geometric mean of immune response related genes (**g**-**i**)

To investigate similarities and differences between our newly developed SSPs and the previously published models, we tested correlation between the raw scores and the models (Fig. 5a-c). Overall, our SSPs were weakly positively correlated with RSS but not with 10-GS.

Cancer cell proliferation is a major biological prognostic determinant in ER+ breast cancer (also largely separating the luminal A from the luminal B subtype), while the immune response has been shown to be important for the prognosis in highly proliferating and ER- breast cancer [18]. To investigate the biology behind the models, we tested correlation between the raw model scores and proliferation and immune response, calculated as the geometric mean of the expression of genes associated with proliferation and immune response, respectively (details in Additional file 2). Overall, our SSPs were weakly correlated with proliferation, but not immune response (Fig. 5d and g). RSS was also weakly correlated with proliferation and weakly negatively correlated with immune response (Fig. 5e and h). 10-GS, on the other hand, was more strongly negatively correlated with both proliferation and immune response (Fig. 5f and i). Further, stratified for ER and RT, the SSPs developed in ER+ tumors correlated with proliferation and weakly with immune response. Conversely, the SSPs developed in ER- tumors negatively correlated with immune response, but did not correlate with proliferation (Additional file 8: Figure S4).

## Discussion

In this study, we developed and validated single-sample predictors (SSPs) that were prognostic for IBTR using a targeted gene expression panel applicable to samples of lower RNA quality. We presented a conceptual idea of applying the SSPs to stratify patients into treatment groups with promising potential. Two previously published radiosensitivity signatures [8, 12] were also tested in our data, and their performance was found to be ER status dependent, which may be explained by the biology behind the different models.

The treatment of primary breast cancer is highly individualized, and tests are available to guide the use of adjuvant endocrine therapy, chemotherapy and anti-HER2 treatment [37, 38]. However, no test is available to guide the use of adjuvant RT, which remains an urgent unmet clinical need. Several attempts have been made towards this aim, but no test has been introduced in clinical use. The reasons are mainly due to lack of follow-up studies and validation, the inability to handle samples of lower RNA quality, which is typical under clinical conditions with FFPE samples, and the models being cohort dependent. We here present a novel approach that aims to overcome these problems, and move individualized RT closer to clinical use. First, we build on previous biological knowledge by including genes that have been previously described in the literature to be associated with radioresistance, in addition to our newly discovered set of genes. Our final SSP models consist of genes from these different sources, and are highly prognostic for IBTR, both in our validation data and in independent public data. In addition, the targeted assay includes genes from two previously described radiosensitivity signatures, giving us an opportunity to validate a surrogate score for these two profiles, which indeed validated our data for prognostication in certain subgroups. Importantly, the 10-GS is also treatment predictive for RT. Second, most clinical samples are handled and stored as FFPE tissue, and an assay able to process RNA extracted from FFPE samples would greatly facilitate its use in the clinical routine. Here, we have used the Nanostring nCounter platform for our targeted assay, which has shown good performance in FFPE samples and is FDA approved for such use with the ProSigna assay [39], and we validated our targeted radiosensitivity panel in samples of lower RNA quality. Although not yet directly tested in FFPE samples, our samples of lower RNA quality are similar to RNA extracted from FFPE samples in terms of the RNA integrity number (RIN) value and fragment length (data not shown). Third, we used a machine learning algorithm, (k-TSP), which relies only on the relative expression of genes within a sample, which should in theory make it both platform and cohort independent. Indeed, we validated the SSPs in data from samples that were partly degraded and in fresh-frozen tumor cohorts, without any scaling or other measure to make the data comparable.

Further, the aim of a radiosensitivity predictor in early breast cancer is to stratify patients and offer treatment only to patients in whom RT had a clinically significant effect. However, patients that do not benefit from RT after BCS may either be those that have the least aggressive tumors, and remain recurrence-free even without RT (requiring de-escalation of treatment), or those with the most aggressive and radioresistant tumors (requiring escalation of treatment). This may complicate the analysis, since those two groups of tumors most likely are not similar in their transcriptomic profiles. The strength of this study is therefore that we developed classifiers that incorporate those two different settings, for not benefitting from RT in treatment stratification, creating three groups for treatment stratification. The results were highly significant in the validation cohort, although we acknowledge the small sample sizes, and the requirement for further validation in larger cohort studies or randomized trials.

However, although we herein showed reproducible classifiers for IBTR prognostication and RT treatment stratification, it must be noted that RT is an effective treatment, with good cost-effectiveness, and relatively mild side effects, which increases the threshold for withholding RT in patients. High predictive accuracy is required from any radiosensitivity predictor for it to be clinically useful. Although promising, the performance of our proposed SSPs and the previously published profiles show that they are not yet ready for clinical use. Validation in additional cohorts may be a next step, but further classifier development is likely needed. Indeed, our SSPs were intentionally trained with default settings using the majority of genes in the panel as a proof of concept. There is great potential to further optimize the model by e.g. reducing the number of gene pairs, weighting the gene pairs, etc. For a final clinical decision tool, one alternative may be to include additional parameters in the models, i.e. combining gene expression data with clinicopathologic variables, intrinsic subtype, and other molecular data into mixed classifiers. Indeed, combining gene expression data with additional information has already been suggested [16, 40]. However, this dataset, especially after the validation of a locked profile, is not sufficient for extensive classifier optimization or evaluation of other clinicopathologic variables.

One limitation of our study is the case-control sampling, meaning that RT was not administered in a randomized fashion. This limits the analyses that can be performed, and e.g. the proposed method of using a Cox model with an interaction term between treatment and gene expression is not feasible in this dataset [41]. Further, the cohort is enriched for patients with IBTR, and thus the Kaplan-Meier curves and HR estimates presented are not representative of the risk of recurrence in a matched population, and should only be interpreted as an indicator of how the different models perform in the specific datasets. The problem of treatment given in a non-randomized fashion is not unique to our dataset, but is a general problem in the development of a RT predictive gene expression signature. The publicly available datasets analyzed here were also non-randomized for RT, and the dataset presented by van de Vijver included patients who underwent both modified radical mastectomy and BCS, while the dataset by Servant et al. contained only patients who underwent BCS. Also, in the publicly available datasets the proportion of patients given RT differs. In the dataset of Servant et al., all patients were given RT, while this was not the case in the van de Vijver et al. cohort. This may explain the observed differences between the datasets when we validated our SSPs. Further, systemic adjuvant treatment was allowed in our study and was not specified in the inclusion criteria, which may introduce bias and make interpretation of the classifier performance difficult in relation to another cohort. Indeed, there are differences in the proportion of chemotherapy and endocrine therapy given in the discovery and validation cohorts (Table 1, Additional file 9: Table S2). However, to correct for this, we performed multivariate Cox regression adjusting for tumor characteristics (subtype, size and positive lymph nodes) and treatment (endocrine therapy and chemotherapy) for both the prognostic SSPs, and the consecutive use of SSPs to stratify patients for treatment, which did not alter the main findings (Additional file 2).

We chose to develop different models for ER+ and ER- breast cancer, as ER status is a major determinant of breast cancer biology [42]. Indeed, when we analyzed the previously reported RSS and 10-GS signatures, they did not perform uniformly for ER+ and ER- disease. To that end, we investigated the biological basis behind the models, focusing on proliferation and immune response, which have been described as the major drivers of breast cancer biology [18]. As our SSPs developed in ER+ breast cancer were correlated with proliferation, one might suspect that we found the difference between luminal A and luminal B tumors, which is defined mainly by proliferation, and that our high-risk tumors were mainly luminal B tumors. However, the rate of high-risk and low-risk predictions was similar in the luminal A and luminal B tumors. Although the performance of the SSPs were slightly higher in the luminal A tumors, the difference was not significant. Furthermore, multivariate modeling including subtype did not alter the findings (Additional file 2). RSS was also correlated with proliferation, and it was trained in a cohort with mainly ER+ tumors all treated with RT, which may explain why it could only be validated in ER + RT+ patients. More interestingly, the 10-GS could only be validated in ER-RT+ patients, and the ER + RT- tumors predicted as radioresistant actually had a lower risk of IBTR, which is consistent with the follow-up study by the original authors [16]. As the 10-GS is negatively correlated with proliferation and immune response, as was also shown recently by the original authors [17], this means that the tumors predicted as radioresistant were mainly slowly proliferating, and it therefore makes sense that ER+ tumors predicted as radioresistant have a better outcome. Further, the tumors predicted as radioresistant have a lower immune response, which may explain why ER- tumors predicted as radioresistant have a worse outcome, as the immune response is more important in highly proliferating and ER- tumors.

## Conclusion

In conclusion, we developed and validated single-sample predictors based on a targeted radiosensitivity gene expression assay using the Nansotring nCounter platform. We validated our SSPs in samples of lower RNA quality, and in external data, with promising results in the treatment stratification of patients. Previously published profiles were also validated in our data, but their performance was highly dependent on the ER status of tumors. Explanations for the difference in performance may be found in the biological basis behind the different classifiers, and should be incorporated in future studies.

## Additional files

Additional file 1: **Figure S3.** Principle component analysis (PCA) plot of the gene expression data from the targeted panel, with coloring for the biobank center from which the samples were derived. Center 1 and 3 had samples of higher quality RNA and constituted the discovery cohort. Center 2 constituted the validation cohort. (PDF 184 kb)

Additional file 2: Supplemental methods, results and discussion. (DOCX 91 kb)

Additional file 3: **Table S1.** Genes included in the targeted 248-gene panel. (CSV 38 kb)

Additional file 4: **Table S3.** Genes in the k-top scoring pairs predictors. (XLSX 24 kb)

Additional file 5: **Figure S1.** Selection of top discrimination genes in the Illumina discovery cohort data. Number of genes in the random forest models are plotted against performance of classifying cases and controls, as measured by cross-validated area under the curve (AUC). The analysis was stratified for estrogen receptor (ER) status and radiotherapy (RT) treatment, and with added patients from other strata, based on a biological rationale as described in the text. (ZIP 171 kb)

Additional file 6: **Figure S2.** Hierarchical clustering of the top discriminating genes selected in the discovery analysis. Genes are presented as rows, and samples as columns. Colors of the columns represent group after stratification for estrogen receptor (ER) status and radiotherapy (RT), with red representing tumors with later ipsilateral breast tumor recurrence (IBTR, cases). Colors of the rows shows the group in which the gene was selected. Each of the main four clusters were compared with the clusters described by Fredlund et al. and the cluster with the highest association has been marked. (PDF 1308 kb)

Additional file 7: **Table S4.** Univariable and multivariable Cox -models for the ER+ tumors including variables of “Give RT” vs “No RT” and “Give more treatment”, radiotherapy, and the interaction term between the prediction variable and RT. (XLSX 8 kb)

Additional file 8: **Figure S4.** Correlation of SSP scores with proliferation and immune response. Raw SSP scores are plotted against a proliferation score and an immune score, respectively. SSP scores are calculated based on the four different models developed stratified for estrogen receptor (ER) status and radiotherapy (RT) (ER+RT+, ER+RT-, ER-RT+, ER-RT-). Pearson correlation values and p-value from a linear model with test for zero slope are plotted together with the linear model fit. (PDF 1160 kb)

Additional file 9: **Table S2.** Patient characteristics per cohort, estrogen receptor status and radiotherapy status. (XLSX 20 kb)

## Abbreviations

10-GS: 10-Gene signature
AUC: Area under the curve
BCS: Breast-conserving surgery
CI: Confidence interval
ER: Estrogen receptor alpha
FFPE: Formalin-fixed paraffin-embedded
GEO: Gene Expression Omnibus
HER2: Human epidermal growth factor receptor 2
HR: Hazard ratio
IBTR: Ipsilateral breast tumor recurrence
k-TSP: k-top scoring pairs
PR: Progesterone receptor
ROC: Receiver operating characteristics
RSI: Radiosensitivity index
RSS: Radiosensitivity signature
RT: Adjuvant radiotherapy
SSP: Single-sample predictor
