# Supplementary figures and images for: Identification and validation of single-sample breast cancer radiosensitivity gene expression predictors
Source: Breast Cancer Res. 2018 Jul 4;20:64. doi: 10.1186/s13058-018-0978-y (PMC6033283; doi:10.1186/s13058-018-0978-y)

Supplemental figure 3

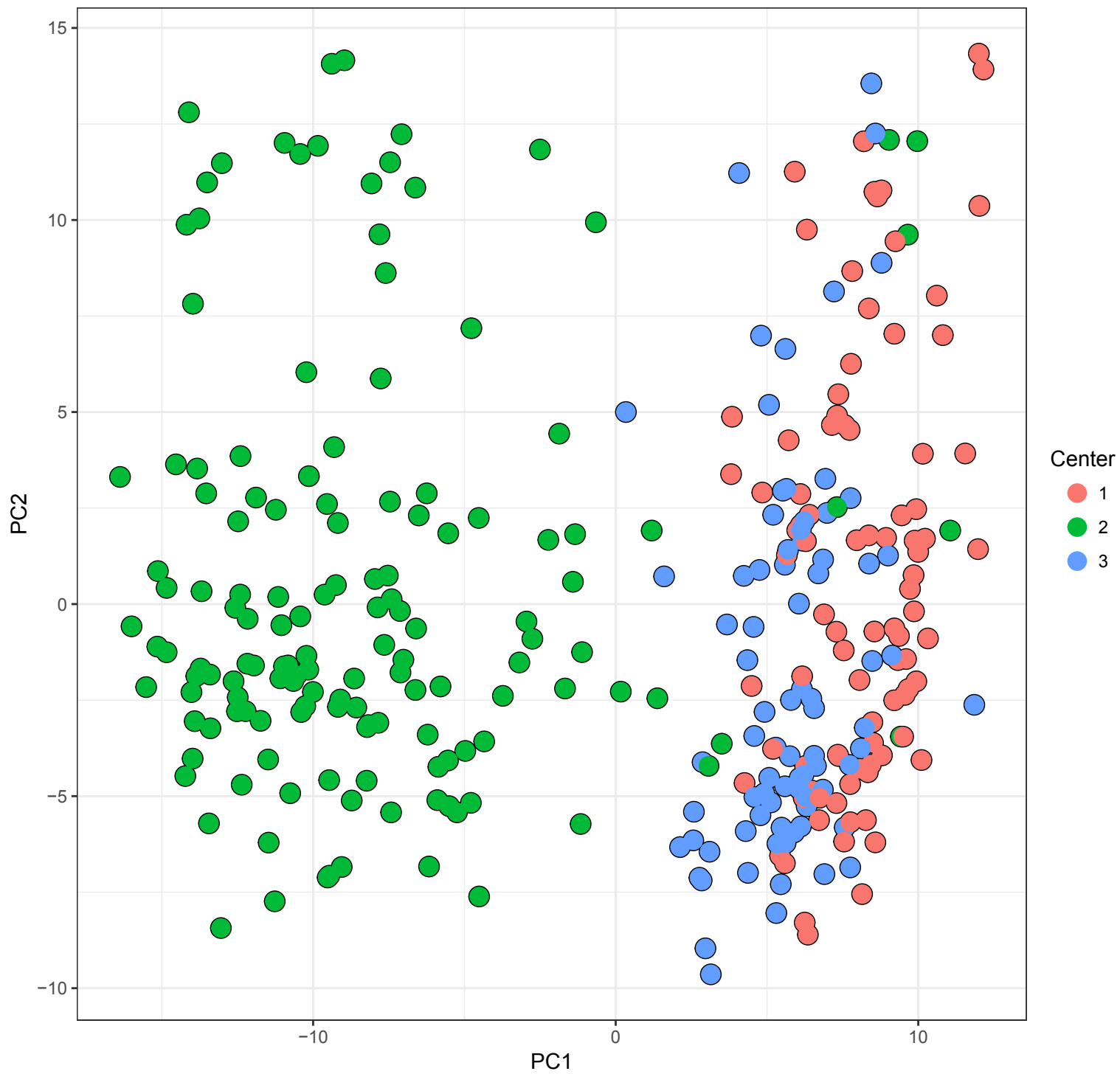

Supplement: Supplementary file 1 — Figure S3. Principle component analysis (PCA) plot of the gene expression data from the targeted panel, with coloring for the biobank center from which the samples were derived. Center 1 and 3 had samples of higher quality RNA and constituted the discovery cohort. Center 2 constituted the validation cohort. (PDF 184 kb) [file 13058_2018_978_MOESM1_ESM.pdf]

Supplemental figure 1A

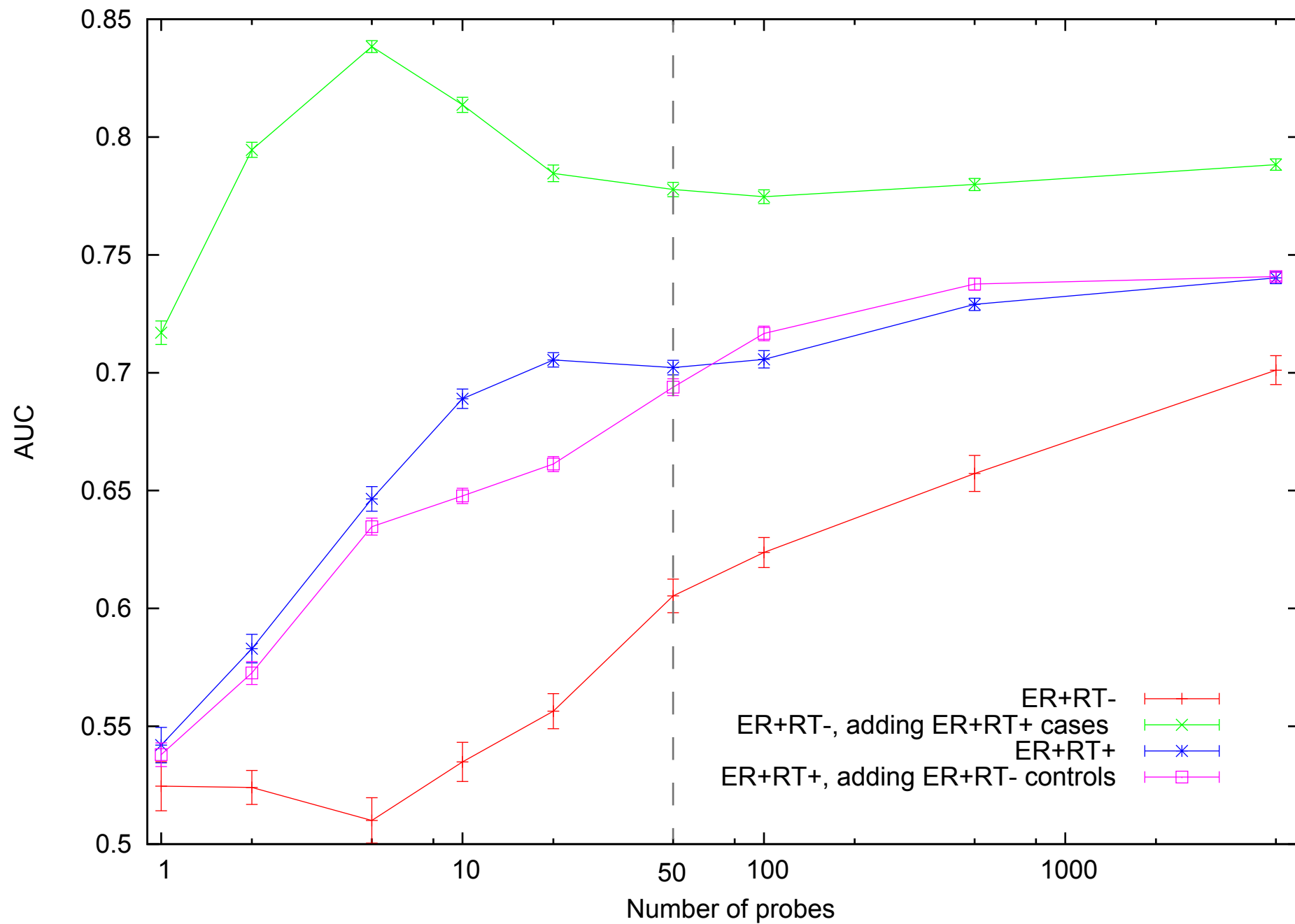

Supplement: Supplementary file 5 — Figure S1. Selection of top discrimination genes in the Illumina discovery cohort data. Number of genes in the random forest models are plotted against performance of classifying cases and controls, as measured by cross-validated area under the curve (AUC). The analysis was stratified for estrogen receptor (ER) status and radiotherapy (RT) treatment, and with added patients from other strata, based on a biological rationale as described in the text. (ZIP 171 kb) [file 13058_2018_978_MOESM5_ESM.zip › Supplemental figure 1A_180306.pdf]

Supplemental figure 1B

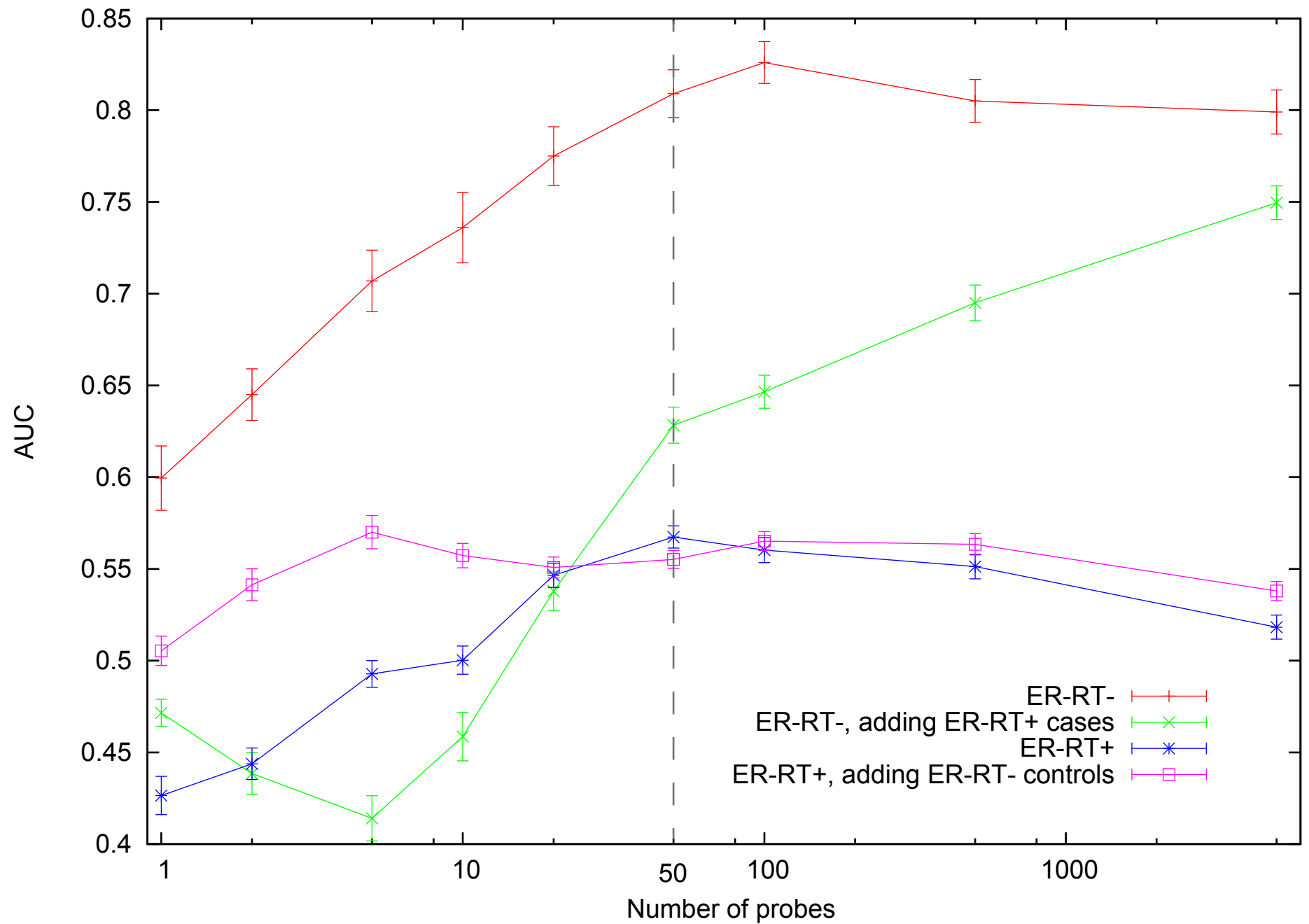

Supplement: Supplementary file 5 — Figure S1. Selection of top discrimination genes in the Illumina discovery cohort data. Number of genes in the random forest models are plotted against performance of classifying cases and controls, as measured by cross-validated area under the curve (AUC). The analysis was stratified for estrogen receptor (ER) status and radiotherapy (RT) treatment, and with added patients from other strata, based on a biological rationale as described in the text. (ZIP 171 kb) [file 13058_2018_978_MOESM5_ESM.zip › Supplemental figure 1B_180306.pdf]
