# Supplementary material for: Identification and validation of single-sample breast cancer radiosensitivity gene expression predictors
Source: Breast Cancer Res. 2018 Jul 4;20:64. doi: 10.1186/s13058-018-0978-y (PMC6033283; doi:10.1186/s13058-018-0978-y)
Supplement: Supplementary file 2 — Supplemental methods, results and discussion. (DOCX 91 kb) [file 13058_2018_978_MOESM2_ESM.docx]

Identification and validation of single sample breast cancer radiosensitivity gene expression predictors

**Martin Sjöström, Johan Staaf, Patrik Edén, Fredrik Wärnberg, Jonas Bergh, Per Malmström, Mårten Fernö, Emma Niméus and Irma Fredriksson**

# Supplemental methods, results and discussion

## Patient data and sample handling

### Samples

Samples were collected from biobanks at three sites. In total, 336 primary tumors were collected and included in the study. Tumors were put directly on ice at the time of surgery and then stored in -80°C until extraction (one region), or stored at -80°C and shipped on dry ice under frozen conditions (two regions). All samples were stored as fresh frozen samples, and at one center additional embedding was made for cryosectioning (TissueTek O.C.T., Sakura Finetek, Ca, USA).

### RNA extraction

RNA extraction was performed at one center. Tissue was cut under frozen conditions and approximately 30 mg of tissue were taken for RNA extraction. An adjacent piece, or imprint in cases where not enough tissue for embedding was available, was taken for microscopy and evaluation of cancer content at the center performing RNA extraction. Samples without cancer cells were excluded. The tissue piece was lysed and homogenized using a TissueLyser, lysis buffer and QIAshredder columns, according to the manufacturer’s instructions (Qiagen, Hilden, Germany). RNA was extracted using the AllPrep mini kit on the QIAcube for samples not embedded for cryosectioning and the RNEasy lipid tissue kit for samples that had been embedded for cryosectioning, according to the manufacturer’s instructions (Qiagen).

### RNA quality control

The amount, concentration and quality of the extracted RNA was tested using a Bioanalyzer 2100 instrument (Agilent Technologies, CA, USA), NanoDrop ND-1000 spectrophotometer (Thermo Fisher Scientific, MA, USA) or Caliper HT RNA LabChip (Perkin Elmer, MA, USA).

### Creation of discovery and validation cohort

When analyzing the quality of the RNA, it was apparent that one center had lower quality than the two other centers (Figure S3). The samples had been transported on dry ice from the biobanks to the center where the extractions were performed, and the reason for the lower quality is believed to be the thawing, freezing and transportation. Since the degraded samples, similar to formalin-fixed paraffin-embedded (FFPE) samples, requires targeted or more robust gene expression analysis, we decided to use the high-quality samples as a discovery cohort for gene discovery and classifier creation (N=172), and the degraded samples as a validation cohort for a targeted assay (N=164). The RNA quality of the discovery cohort was higher with a median RIN value of 8.0 (range 5.2-9.8) and was lower for the validation cohort with a median RIN value of 2.6 (range 0-7.7).

## Discovery cohort and Illumina microarray gene expression analysis

### Gene expression analysis

The discovery cohort consisted of 172 primary tumors from two centers. Samples were run with the Illumina HT12 v4 microarray according to manufacturer’s instructions (Illumina, San Diego, CA). Analyses were performed by the Swegene Centre for Integrative Biology at Lund University (SCIBLU Genomics). The input was 575ng and the hybridization was performed on three plates. Tree samples were replicated across hybridization plates and one RNA standard was included for comparison.

### Preprocessing of Illumina HT12 v4 data

Raw summary level data was exported using BeadStudio (Illumina). The data was then normal-exponential background corrected, quantile normalized, and log 2 transformed using the limma R package [1]. An offset of 16 was applied to avoid negative values, as per default settings and as previously recommended [2]. An annotation package for the quality of individual probes was used, and probes annotated as “bad” or “no match” were discarded [3]. Further filtering was made using the detection p-value calculated by Illumina BeadStudio (Illumina), and probes were required to have a detection p-value < 0.01 in at least 80% of samples, or to have a high expression in the fewer than 80% samples expressing the probe with p < 0.01 (normalized log2 intensity > 7), as we hypothesized that probes highly expressed in a minority of samples could still be of biological importance, and should thus be included. Potential batch-effects from biobank center and hybridization plate were corrected with combat using the sva R package with default settings and no “variable of interest” [4]. A variance filter was then applied to select the 5000 probes with highest variance, which were used for further analysis.

### Top discriminating genes

The analysis to find top discriminating genes between tumors with or without later ipsilateral breast tumor recurrence (IBTR) was performed stratified for estrogen receptor (ER) status and radiotherapy (RT), creating four different subgroups (ER+RT+, ER+RT-, ER-RT+, ER-RT-). The rational for the stratified analysis was the large biological difference between ER+ and ER- disease [5], and the ER status dependent performance of previous classifiers, as reported in the literature [6]. Further, finding RT related genes in the adjuvant setting in breast cancer is complicated by that fact that the lack of effect form RT could either be because the tumor intrinsically lacks the ability to recur, or that it is radioresistant. In the lack of a randomized study, we hypothesized that the RT- group could be used to find genes associated with the ability to recur without RT, and the RT+ tumors to find genes associated with radioresistance. Thus, the discovery analysis was focused on finding four different sets of genes. However, one could also argue that RT- patients without an IBTR could be a control also in the RT+ treated group, and conversely, that RT+ patients with an IBTR could be a case also in the RT- group. This would enhance the number of patients and the statistical power in the analysis, and we also analyzed these groups.

In each of the groups, top discriminating genes among the 5000 genes filtered for further analysis were analyzed with a machine learning approach utilizing a random forest model in a double validation loop. In an inner bagging loop, samples were chosen randomly (stratified, with replacement) and a random forest predictor was trained. The importance of the individual genes was tested on the out-of-bag samples, and the mean importance of the baggings was calculated. New predictors based on different numbers of genes allowed in the models were validated with an outer cross-validation loop, in order to select the number of genes to use in each model. Area under the curve (AUC) in a receiver operating characteristics (ROC) analysis was used as the measurement of performance. The number of trees in each random forest was set to 10,000, and we used 10-fold cross-validation repeated 5 times. The whole procedure was repeated 20 times to make error estimates (Figure S1). The analysis was based on the caret R package with in-house modifications [7].

Based on these analyses, we chose to include 50 genes from the ER+RT+, ER+RT- and ER-RT- groups, but no genes from the ER-RT+ group since the performance never exceeded AUC 0.56. In addition, we added 20 genes from the ER+RT- group with added ER+RT+ cases, as the larger number of patients appeared to increase the performance. In total, due to overlap and multiple probes from the same gene, 155 genes were selected for inclusion in a targeted assay.

### Biology of the selected genes

The top discriminating genes were selected based on their performance in the models, and no functional or biological filtering was made at this point. To get an overview of the biology behind the selected genes, we did a hierarchical clustering of the genes, and investigated the association of the main clusters with the clusters described by Fredlund et al. using the freely available tool GOBO (Figure S2) [8, 9]. As expected, proliferation and immune response genes were picked up, with proliferation genes being most important in ER+ and immune response genes more important in ER- tumors. However, a large proportion of genes were weakly associated with the clusters described by Fredlund et al., and one cluster did not correlate with any of the Fredlund clusters, suggesting potentially RT-associated specific genes.

## Creation of a targeted gene expression panel

### Selection of genes

To the 155 genes selected for further analysis in the discovery cohort, we added genes from the three most recently described radio resistance gene expression profiles in breast cancer: the radioresistance index (RSI) containing ten genes, the radiosensitivity signature (RSS) with 51 genes, and the seven genes described by Tramm et al. [10-12]. We further added genes from the literature that have been described to be associated with biology relevant to radioresistance or risk of IBTR in breast cancer (Table S1). Among the genes were genes associated with apoptosis (*BCL2*) [13], DNA-repair (*BRCA1*, *BRCA2* and survivin/*BIRC5*) [14, 15], the MET-HGF pair [16], hypoxia (*HIF1* and *HIF2*) [17] and *WRAP53* [18]. We also added genes important for breast cancer biology or subtyping *(ESR1, PGR, ERBB2, MKI67, AURKA, FOXC1*). Finally, we added thirteen housekeeping genes previously used by Nanostring in their targeted gene expression assays (Table S1). In total, 248 genes were selected for the development of a targeted assay.

We chose to use the Nanostring nCounter platform (Nanostring Technologies, Washington, USA) for the targeted assay, as the platform has been proven to handle samples of low RNA quality, including FFPE samples, and is already FDA approved for breast cancer analysis with the Prosigna assay [19]. Probe design was performed by Nanostring and custom codesets with the XT-formulation were produced and shipped ready for analysis by Nanostring. Details of the probes are found in Table S1.

## Gene expression analysis with a targeted Nanostring nCounter assay

### Gene expression analysis

336 primary tumors (172 from the discovery cohort and 164 from the validation cohort) were analyzed with the targeted assay. Samples were run in a randomized order. Analysis was performed according to the manufacturer’s instructions using the Nanostring prep station and digital analyzer. Input amount was 100ng for samples with RIN ≥ 6.0 and 200ng for samples with RIN < 6.0, as the quality of RNA affects fragment length and the number of molecules detected, and Nanostring recommends increasing the input for FFPE samples.

### Preprocessing of the data

Quality filtering of the probes was applied based on negative control probes. Probes expressed above the negative controls (geometric mean) in less than 80% the samples were removed from further analysis. This affected 7/248 probes (*AKNA*, *KCNMB2*, *OR8G2*, *hcg2023290*, *GCSH*, *PRAME* and *MYOG1*). Samples were then normalized based on the positive control probes and a normalization factor calculated as the average geometric mean of positive controls divided by the sample specific geometric mean of positive controls. As a quality filter for technical problems, samples with positive normalization factors <0.3 or >3 were removed, excluding four samples (three from the discovery set and one from the validation set). The data was further normalized based on housekeeping genes and the seven most stable housekeeping genes from analysis with the NormqPCR package were used (*MRPL19, PUM1, SF3A1, PSMC4, HPRT1, ACTB, PGK1*) [20]. A normalization factor was calculated as the average geometric mean of the seven housekeeping genes divided with the geometric mean of the seven housekeeping genes of each sample. As a quality filter for sample problems, samples with normalization factors <0.1 or >10 were excluded (25 samples were excluded, one from the discovery cohort and 24 from the validation cohort). Finally, log2 transformation was performed. For the final analysis, 168 samples were used from the discovery cohort and 139 from the validation cohort.

## Development of single sample predictors (SSPs)

As expected, there were major batch effects between the discovery cohort and the validation cohort (discovery cohort was samples from center 1 and 3, validation cohort was samples from center 2, Figure S3). For this reason, and for platform independency (as stated in the main text), we chose to develop single sample predictors (SSP) that only uses information on the relative intensities within a sample. Model training was performed with the switchbox R package, that creates simple rules based on expression of pairs of genes [21]. Default settings were used, which selects the optimal number of genes by the rank-in-context method and cross-validation in the discovery cohort,[22] and the majority vote as cut point for classifying a sample as a case/high risk. All genes in the panel were allowed in the models and the minimum number of pairs was set to 100, meaning 200 genes and thus a combination of previously described genes and novel genes from our discovery analysis, as previous studies have shown that larger number of genes produces more stable gene signatures [23]. Full details of the genes included and pairwise combination is provided in Table S3. The SSPs are defined such as the first gene in the pair is on average lower than the second in the low-risk samples. When classifying samples, the algorithm gives a point if the first gene is higher than the second, meaning that a high score is associated with higher risk of recurrence.

The analysis was stratified for ER status and RT, producing four different classification problems and four different models (ER+RT+, ER+RT-, ER-RT+, ER-RT-). The models were trained and locked in the discovery cohort and then tested in the validation cohort. All presented results are from the validation cohort. To measure the performance of our models, we used a receiver operating characteristics (ROC) analysis with the area under the curve (AUC) as outcome using the pROC R package [24]. Further, we did Kaplan-Meier survival analysis, calculated the p-value with a log-rank test, and performed Cox regression modeling, using the survival R package [25]. Important to note is that the present study is not a cohort study, and enriched for patients with later IBTR, and thus the Kaplan-Meier estimates, or Cox hazard ratios, cannot be interpreted as the risk in a general population, but only as an indicator on how the models perform in our dataset. The same is true for the two public datasets analyzed.

The difference between the discovery cohort (higher quality RNA) and the validation cohort (lower quality RNA) was apparent from a principal component analysis (Figure S3). Instead of trying to adjust the data, which could make a classifier potentially cohort and platform dependent, we chose to use SSP models based on a k-TSP algorithm. Since the SSPs only rely on relative expression within a sample, it should in theory be normalization and platform independent. It is also conceptually a much simpler model than more advanced machine learning algorithms, such as random forests and support vector machines, which facilitates biological interpretation. However, two aspects may be problematic. First, the RNA degradation in our validation cohort, and the degradation in FFPE samples, may not be uniform across all genes. Rather, the opposite has been suggested; it seems some genes are seems to be more prone to degradation than others [26]. A classifier that relies on relative intensities of genes can be vulnerable if the relative intensities shifts based on degradation, and not on biology. Second, the variance and expression level distribution of the genes must allow the genes to be ranked differently. I.e. it is not enough that a gene can separate patients by high or low expression, there must also be a shift in expression compared to another gene. Ultimately, the value of a model must be tested in independent data. We were able to validate our SSPs trained in high quality samples in degraded samples as well as in public data sets, supporting the rational to use this type of model. Furthermore, the previously published profiles were tested in all samples, as well as separately in the discovery and validation cohort, with results being largely similar indicating that the analysis of degraded samples with our targeted assay was successful (data not shown).

## Validation in public datasets

To test our models in two public datasets [27, 28] we mapped the genes in our SSP models to the probes in the respective datasets. If there were more than one probe per gene, the probe with the highest variance was used. Not all genes could be mapped. In the datasets from Servant et al., three genes could not be mapped (*IGKC, RELL1* and *WFDC21*), and in the dataset from van de Vijver et al., 34 genes could not be mapped. (*ACCS, C11ORF52, CBX2, DCBLD1, DENND1B, DSG2, GLRX3, GPSM3, HBA1, HELLS, HGF, HLA-DQA2, HS.445414, IGKC, LGALS9, LOC100630918, LOC196752, LOC646567, LOC653080, MBOAT1, MYO15B, NRROS, OSTC, PGAM1, RELL1, RELA, SERPINE2, TMEM191B, TOMM5, TPK1, WFDC21P, ZFP14, ACTB, TUBB*). To be able to test our SSP models, they were used without the pairs containing these genes. In case of missing data points (<1%) in the data set by van de Vijver et al., imputation with the mean expression was used.

## Testing previously published signatures

The radiosensitivity signature (RSS) was calculated as described in the original publication[11]. Our data was median centered per gene, and the expression value was scaled to be between -2 and 2.

A surrogate score for the radiosensitivity index (referred to as 10-GS) was calculated as previously described [10]. In the original publication, the 25^th^ percentile was used to dichotomize the patients into radioresistant and radiosensitive tumors, based on the assumption that roughly 25% of tumors in a population are radioresistant. In the present study, we have enriched for patients with a later IBTR and the rate of recurrence is higher, and we therefore used the median as the cut point to define a tumor as radioresistant or radiosensitive.

We were not able to recreate the model described by Tramm et al. from the description in their paper, and thus we included the genes in our panel, but did not calculate their “CVSI” score.

## Correlation of SSP, RSS and 10-GS

We investigated the correlation between the three different signatures by plotting the raw scores from each signature against the others using both our discovery and validation cohorts. R correlation values were calculated and the p-value with a linear regression with test for zero slope.

## Biology of the signatures

To investigate the biology behind the signatures, we did a correlation with two of the most important biological determinants of prognosis in breast cancer: proliferation of the tumor and immune response, in the combined discovery and validation data from the targeted radiosensitivity panel. A proliferation score was calculated as the geometric mean of two proliferation genes (*MKI67* and *AURKA*). To create an immune score, we did a search of our genes in the Gene Ontology tool PANTHER [29], and calculated the geometric mean of genes flagged as part of the immune response (*IRF1, IGKC, STAT1, OSMR, CCL19, RelA, IRF8, FGR, TNFRSF1B, C3*). The raw scores from the three signatures (SSP, RSS, 10-GS) were then plotted against the proliferation and immune scores, Pearson correlation was calculated, and p-values were calculated with a linear regression with test for zero slope. For the SSPs in Figure 5, the samples were classified with the corresponding classifier according to ER and RT status and the results presented in a single graph for space reasons.To further clarify the biology in different groups, we also present the same analysis stratified for ER status and RT, in Figure S4. Here, for each SSP model, we calculated the score for all samples in the discovery cohort and the validation cohort, and correlated the raw scores with proliferation scores and immune scores, respectively (Figure S4).

## Luminal A and Luminal B tumors

As the signatures developed in the ER+ tumors seemed to be correlated with proliferation, we tested if our models were selecting for subtype.Since the full gene set to subtype tumors according to multigene classifiers was not available for all tumors, a surrogate subtyping with ER, progesterone receptor (PR), HER2 and Ki67 was used. Receptor and Ki67 status from the pathology laboratory report was used if available. For tumors with missing pathology data, gene expression values were used and the cut point was based on a comparison of the gene expression values of tumors with pathology data. Luminal A tumors were defined as ER+, PR+, HER2- and Ki67 low, Luminal B tumors as ER+, HER2- and PR- or Ki67 high, HER2+ tumors asHER2+ and any ER status and Ki67 status, thus combining luminal and non-luminal HER2 tumors. Triple negative tumors were defined as ER-, PR- and HER2-.

Of the Luminal A tumors, 44/60 (73%) of tumors were classified as low risk of recurrence and 18/29 (62%) of Luminal B tumors were classified as low risk. Thus, our model does not simply split the samples between Luminal A and B. The performance of the models seemed to be slightly higher in the Luminal A tumors, although the sample size prohibits any final conclusions (Luminal A RT+ AUC 0.71 (95%CI 0.56-0.86), Luminal B RT+ AUC 0.56 (95%CI 0.36-0.81), Luminal A RT- AUC 0.87 (95%CI 0.65-1.0) and Luminal B RT- AUC 1 (95%CI 1.0-1.0).

## Other clinicopathologic variables

To test if our SSP models were independent of other clinicopathologic variables, and thus add information, we created a Cox regression model. We used the dichotomized variable of our models, with the default cut point of the majority vote. In a univariate analysis, our models had an overall hazard ratio of 3.1 (95%CI = 1.9-5.2, p<0.001). When adjusting for chemotherapy, endocrine therapy, positive lymph nodes, subtype and size, the effect was only marginally altered (HR 2.8 95%CI 1.6-5.0, p<0.001), thus supporting that our SSP models are independent of these variables. Similarly, the effect of RT in the treatment groups predicted by applying consecutive SSPs was independent when adjusting for the same variables (“No RT”: univariable HR for RT 0.65 95%CI 0.22-1.9, p=0.46, multivariable HR for RT 0.90 95%CI 0.21-3.7, p=0.88. “Give treatment”: univariable HR for RT 0.03 95%CI 0-0.24, p<0.001, multivariable HR for RT 0.03 95%CI 0-0.48, p=0.01. “Give more treatment”: univariable HR for RT 0.56 95%CI 0.15-2.0, p=0.37, multivariable HR for RT 0.91 95%CI 0.18-4.7, p=0.91). Again, this is not a cohort study and the reported hazard ratios cannot be generalized, but should be interpreted as the performance of the models in this data. Further, this dataset was not collected to analyze the importance of different clinicopathologic variables, and the results from the Cox models presented here should be interpreted with caution.

# Supplemental figure legends

**Figure S1.** Selection of top discrimination genes in the Illumina discovery cohort data. Number of genes in the random forest models are plotted against performance of classifying cases and controls, as measured by cross-validated area under the curve (AUC). The analysis was stratified for estrogen receptor (ER) status and radiotherapy (RT) treatment, and with added patients from other strata, based on a biological rationale as described in the text.

**Figure S2.** Hierarchical clustering of the top discriminating genes selected in the discovery analysis. Genes are presented as rows, and samples as columns. Colors of the columns represent group after stratification for estrogen receptor (ER) status and radiotherapy (RT), with red representing tumors with later ipsilateral breast tumor recurrence (IBTR, cases). Colors of the rows shows the group in which the gene was selected. Each of the main four clusters were compared with the clusters described by Fredlund et al.[8] and the cluster with the highest association has been marked.

**Figure S3.** Principle component analysis (PCA) plot of the gene expression data from the targeted panel, with coloring for the biobank center from which the samples were derived. Center 1 and 3 had samples of higher quality RNA and constituted the discovery cohort. Center 2 constituted the validation cohort.

**Figure S4.** Correlation of SSP scores with proliferation and immune response. Raw SSP scores are plotted against a proliferation score and an immune score, respectively. SSP scores are calculated based on the four different models developed stratified for estrogen receptor (ER) status and radiotherapy (RT) (ER+RT+, ER+RT-, ER-RT+, ER-RT-). Pearson correlation values and p-value from a linear model with test for zero slope are plotted together with the linear model fit.

# References

1. Ritchie ME, Phipson B, Wu D, Hu Y, Law CW, Shi W, Smyth GK: **limma powers differential expression analyses for RNA-sequencing and microarray studies**. *Nucleic acids research* 2015, **43**(7):e47.

2. Ritchie ME, Dunning MJ, Smith ML, Shi W, Lynch AG: **BeadArray expression analysis using bioconductor**. *PLoS computational biology* 2011, **7**(12):e1002276.

3. Dunning M, Lynch A, Eldridge M: **illuminaHumanv4.db: Illumina HumanHT12v4 annotation data (chip illuminaHumanv4)** *R package version 1260* 2015.

4. Leek JT, Johnson WE, Parker HS, Fertig EJ, Jaffe AE, Storey JD, Zhang Y, Torres LC: **sva: Surrogate Variable Analysis. R package version 3.18.0.** 2017.

5. Gruvberger S, Ringner M, Chen Y, Panavally S, Saal LH, Borg A, Ferno M, Peterson C, Meltzer PS: **Estrogen receptor status in breast cancer is associated with remarkably distinct gene expression patterns**. *Cancer research* 2001, **61**(16):5979-5984.

6. Torres-Roca JF, Fulp WJ, Caudell JJ, Servant N, Bollet MA, van de Vijver M, Naghavi AO, Harris EE, Eschrich SA: **Integration of a Radiosensitivity Molecular Signature Into the Assessment of Local Recurrence Risk in Breast Cancer**. *International journal of radiation oncology, biology, physics* 2015, **93**(3):631-638.

7. Max K: **Contributions from Jed Wing, Steve Weston, Andre Williams, Chris Keefer, Allan Engelhardt, Tony Cooper, Zachary Mayer, Brenton Kenkel, the R Core Team, Michael Benesty, Reynald Lescarbeau, Andrew Ziem, Luca Scrucca, Yuan Tang and Can Candan. (2016). caret: Classification and Regression Training. R package version 6.0-68.** In*.*

8. Fredlund E, Staaf J, Rantala JK, Kallioniemi O, Borg A, Ringner M: **The gene expression landscape of breast cancer is shaped by tumor protein p53 status and epithelial-mesenchymal transition**. *Breast cancer research : BCR* 2012, **14**(4):R113.

9. Ringner M, Fredlund E, Hakkinen J, Borg A, Staaf J: **GOBO: gene expression-based outcome for breast cancer online**. *PloS one* 2011, **6**(3):e17911.

10. Eschrich SA, Fulp WJ, Pawitan Y, Foekens JA, Smid M, Martens JW, Echevarria M, Kamath V, Lee JH, Harris EE *et al*: **Validation of a radiosensitivity molecular signature in breast cancer**. *Clinical cancer research : an official journal of the American Association for Cancer Research* 2012, **18**(18):5134-5143.

11. Speers C, Zhao S, Liu M, Bartelink H, Pierce LJ, Feng FY: **Development and Validation of a Novel Radiosensitivity Signature in Human Breast Cancer**. *Clinical cancer research : an official journal of the American Association for Cancer Research* 2015, **21**(16):3667-3677.

12. Tramm T, Mohammed H, Myhre S, Kyndi M, Alsner J, Borresen-Dale AL, Sorlie T, Frigessi A, Overgaard J: **Development and validation of a gene profile predicting benefit of postmastectomy radiotherapy in patients with high-risk breast cancer: a study of gene expression in the DBCG82bc cohort**. *Clinical cancer research : an official journal of the American Association for Cancer Research* 2014, **20**(20):5272-5280.

13. Kyndi M, Sorensen FB, Knudsen H, Alsner J, Overgaard M, Nielsen HM, Overgaard J: **Impact of BCL2 and p53 on postmastectomy radiotherapy response in high-risk breast cancer. A subgroup analysis of DBCG82 b&c**. *Acta oncologica (Stockholm, Sweden)* 2008, **47**(4):608-617.

14. Nilsson MP, Hartman L, Kristoffersson U, Johannsson OT, Borg A, Henriksson K, Lanke E, Olsson H, Loman N: **High risk of in-breast tumor recurrence after BRCA1/2-associated breast cancer**. *Breast cancer research and treatment* 2014, **147**(3):571-578.

15. Vequaud E, Desplanques G, Jezequel P, Juin P, Barille-Nion S: **Survivin contributes to DNA repair by homologous recombination in breast cancer cells**. *Breast cancer research and treatment* 2016, **155**(1):53-63.

16. Veenstra C, Perez-Tenorio G, Stelling A, Karlsson E, Mirwani SM, Nordenskoljd B, Fornander T, Stal O: **Met and its ligand HGF are associated with clinical outcome in breast cancer**. *Oncotarget* 2016, **7**(24):37145-37159.

17. Trastour C, Benizri E, Ettore F, Ramaioli A, Chamorey E, Pouyssegur J, Berra E: **HIF-1alpha and CA IX staining in invasive breast carcinomas: prognosis and treatment outcome**. *International journal of cancer Journal international du cancer* 2007, **120**(7):1451-1458.

18. Garvin S, Tiefenbock K, Farnebo L, Thunell LK, Farnebo M, Roberg K: **Nuclear expression of WRAP53beta is associated with a positive response to radiotherapy and improved overall survival in patients with head and neck squamous cell carcinoma**. *Oral oncology* 2015, **51**(1):24-30.

19. Nielsen T, Wallden B, Schaper C, Ferree S, Liu S, Gao D, Barry G, Dowidar N, Maysuria M, Storhoff J: **Analytical validation of the PAM50-based Prosigna Breast Cancer Prognostic Gene Signature Assay and nCounter Analysis System using formalin-fixed paraffin-embedded breast tumor specimens**. *BMC cancer* 2014, **14**:177.

20. Perkins JR, Dawes JM, McMahon SB, Bennett DL, Orengo C, Kohl M: **ReadqPCR and NormqPCR: R packages for the reading, quality checking and normalisation of RT-qPCR quantification cycle (Cq) data**. *BMC genomics* 2012, **13**:296.

21. Afsari B, Fertig EJ, Geman D, Marchionni L: **switchBox: an R package for k-Top Scoring Pairs classifier development**. *Bioinformatics (Oxford, England)* 2015, **31**(2):273-274.

22. Afsari B, Braga-Neto UM, Geman D: **Rank discriminants for predicting phenotypes from RNA expression**. *The Annals of Applied Statistics* 2014, **8**(3):1469-1491.

23. Lauss M, Ringner M, Hoglund M: **Prediction of stage, grade, and survival in bladder cancer using genome-wide expression data: a validation study**. *Clinical cancer research : an official journal of the American Association for Cancer Research* 2010, **16**(17):4421-4433.

24. Robin X, Turck N, Hainard A, Tiberti N, Lisacek F, Sanchez JC, Muller M: **pROC: an open-source package for R and S+ to analyze and compare ROC curves**. *BMC bioinformatics* 2011, **12**:77.

25. Therneau T: **A Package for Surival Analysis in S. version 2.38**. [*http://CRANR-projectorg/package=survival*](http://CRANR-projectorg/package=survival) 2015.

26. Gallego Romero I, Pai AA, Tung J, Gilad Y: **RNA-seq: impact of RNA degradation on transcript quantification**. *BMC biology* 2014, **12**:42.

27. Servant N, Bollet MA, Halfwerk H, Bleakley K, Kreike B, Jacob L, Sie D, Kerkhoven RM, Hupe P, Hadhri R *et al*: **Search for a gene expression signature of breast cancer local recurrence in young women**. *Clinical cancer research : an official journal of the American Association for Cancer Research* 2012, **18**(6):1704-1715.

28. van de Vijver MJ, He YD, van't Veer LJ, Dai H, Hart AA, Voskuil DW, Schreiber GJ, Peterse JL, Roberts C, Marton MJ *et al*: **A gene-expression signature as a predictor of survival in breast cancer**. *The New England journal of medicine* 2002, **347**(25):1999-2009.

29. Mi H, Huang X, Muruganujan A, Tang H, Mills C, Kang D, Thomas PD: **PANTHER version 11: expanded annotation data from Gene Ontology and Reactome pathways, and data analysis tool enhancements**. *Nucleic acids research* 2017, **45**(D1):D183-d189.

30. Paquet ER, Hallett MT: **Absolute assignment of breast cancer intrinsic molecular subtype**. *Journal of the National Cancer Institute* 2015, **107**(1):357.
