# Supplementary material for: Identification and validation of single-sample breast cancer radiosensitivity gene expression predictors
Source: Breast Cancer Res. 2018 Jul 4;20:64. doi: 10.1186/s13058-018-0978-y (PMC6033283; doi:10.1186/s13058-018-0978-y)

Supplemental figure 2

Hierarchical clustering of the genes included from the discovery analysis

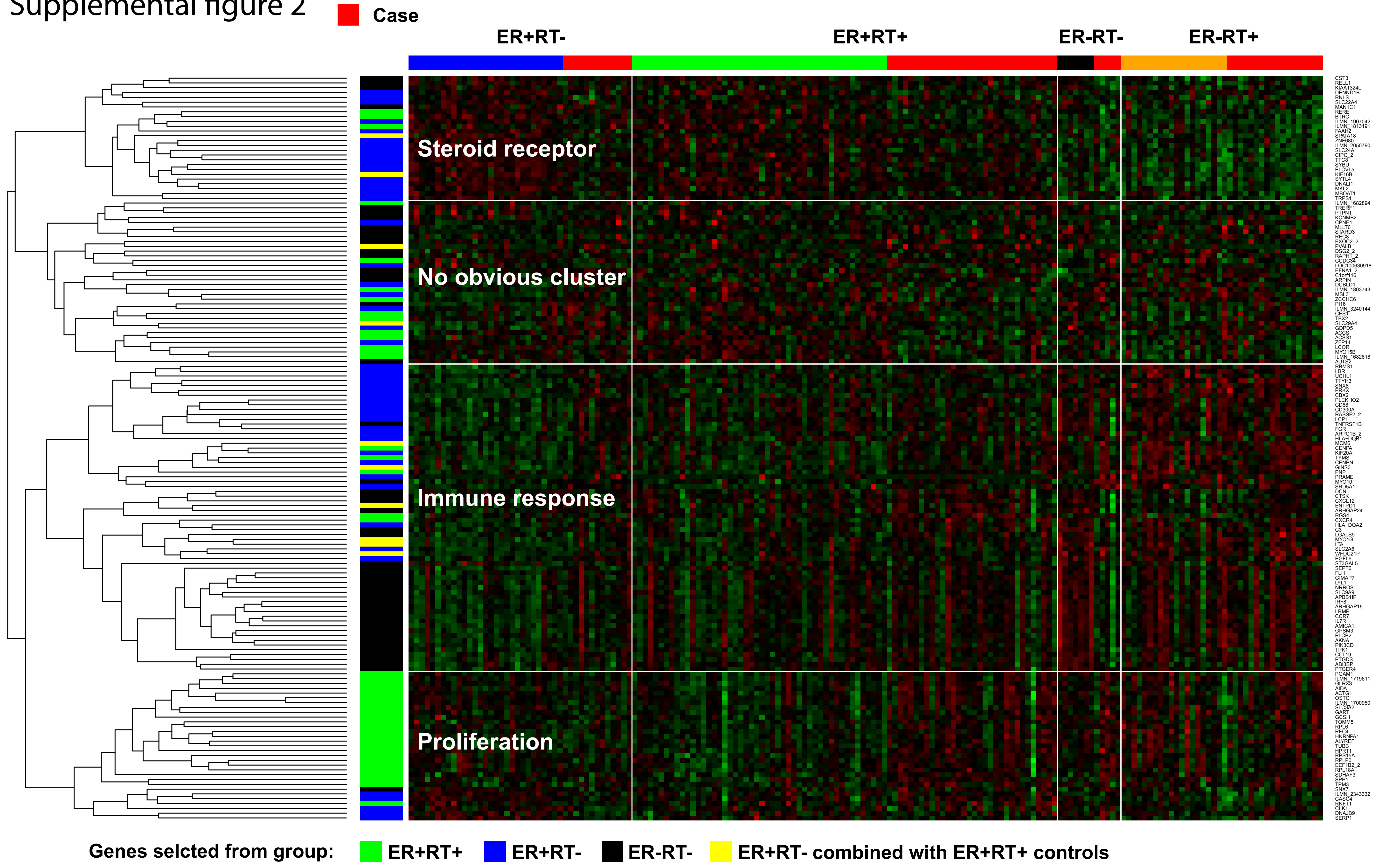

Supplement: Supplementary file 6 — Figure S2. Hierarchical clustering of the top discriminating genes selected in the discovery analysis. Genes are presented as rows, and samples as columns. Colors of the columns represent group after stratification for estrogen receptor (ER) status and radiotherapy (RT), with red representing tumors with later ipsilateral breast tumor recurrence (IBTR, cases). Colors of the rows shows the group in which the gene was selected. Each of the main four clusters were compared with the clusters described by Fredlund et al. and the cluster with the highest association has been marked. (PDF 1308 kb) [file 13058_2018_978_MOESM6_ESM.pdf]
