# Supplementary material for: Identification and validation of single-sample breast cancer radiosensitivity gene expression predictors
Source: Breast Cancer Res. 2018 Jul 4;20:64. doi: 10.1186/s13058-018-0978-y (PMC6033283; doi:10.1186/s13058-018-0978-y)

Supplemental figure 4

Correlation of SSP scores with proliferation and immune response

ER+RT+

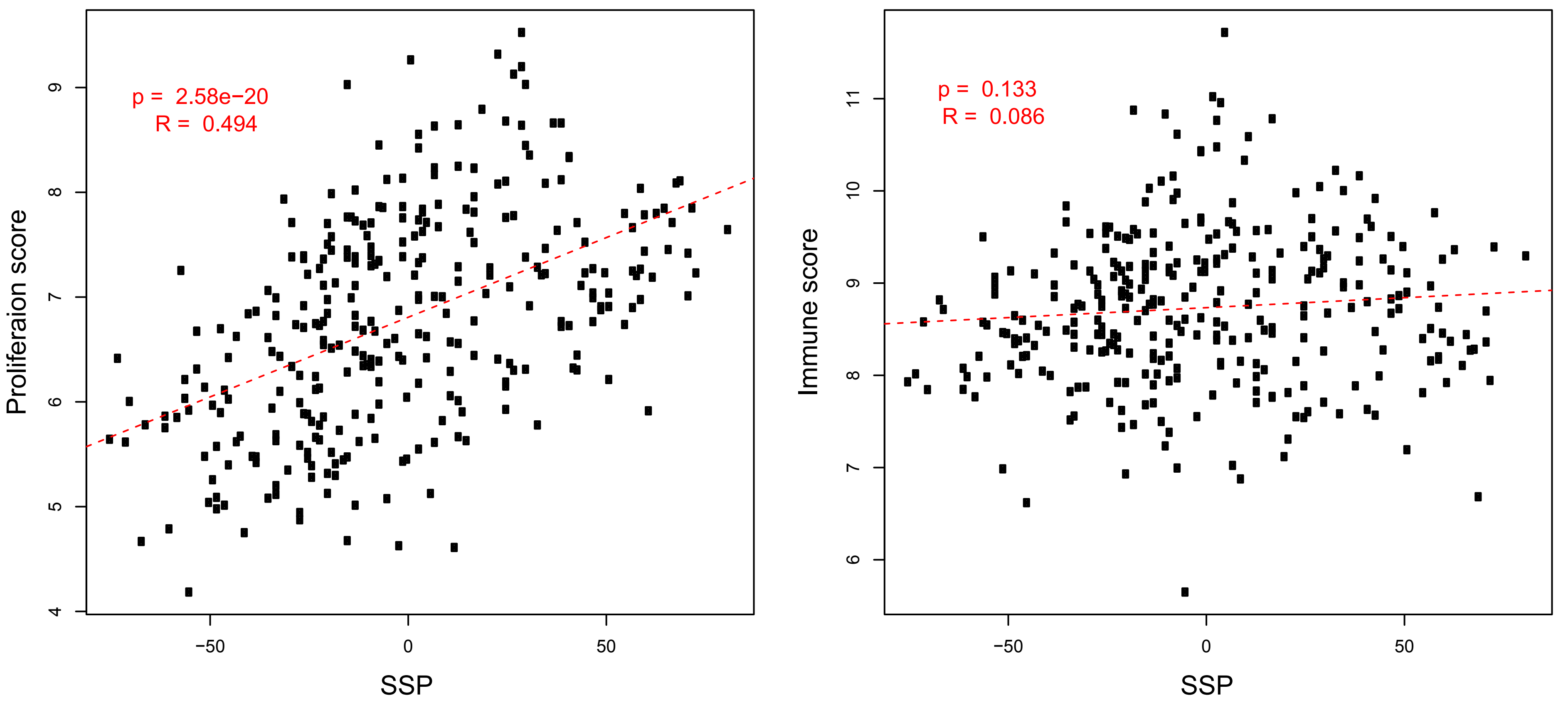

ER+RT-

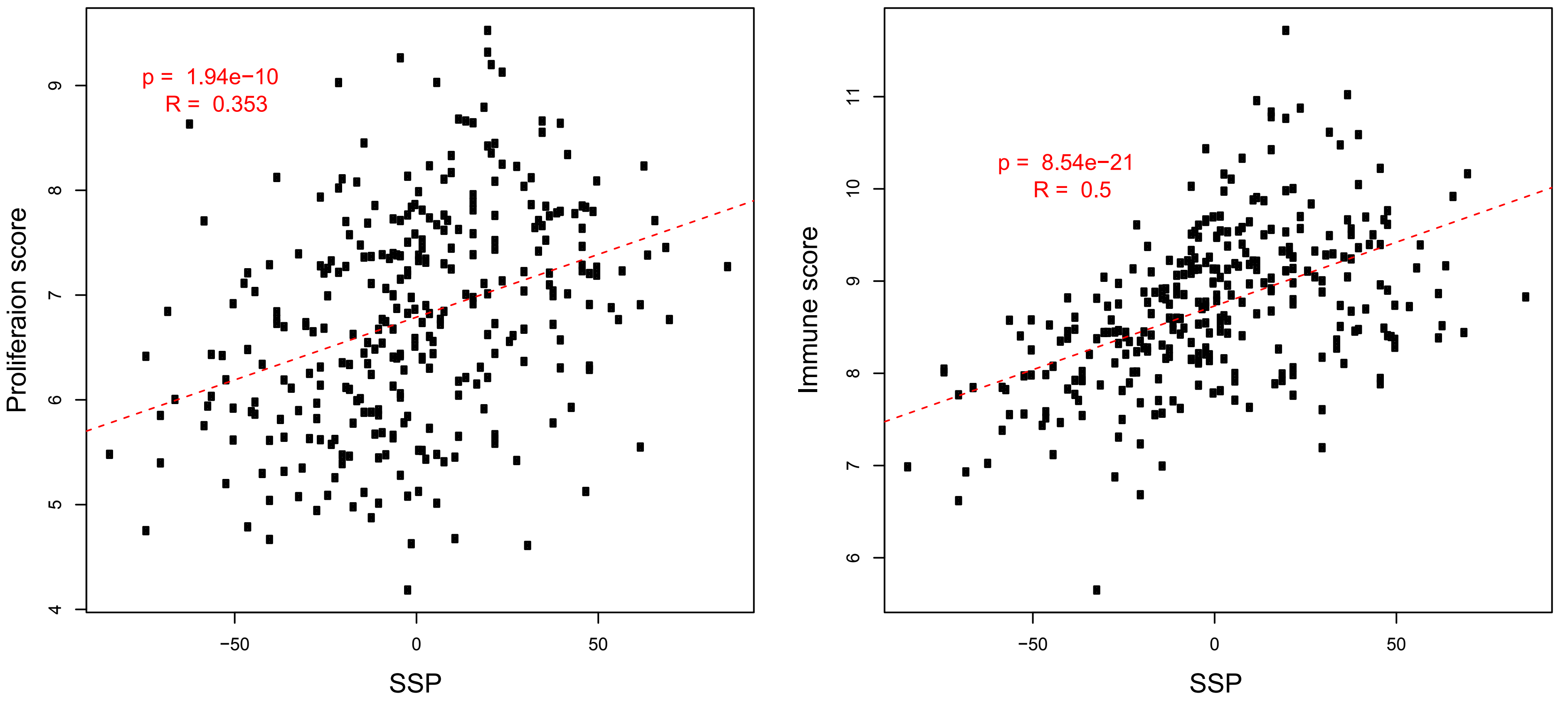

ER-RT+

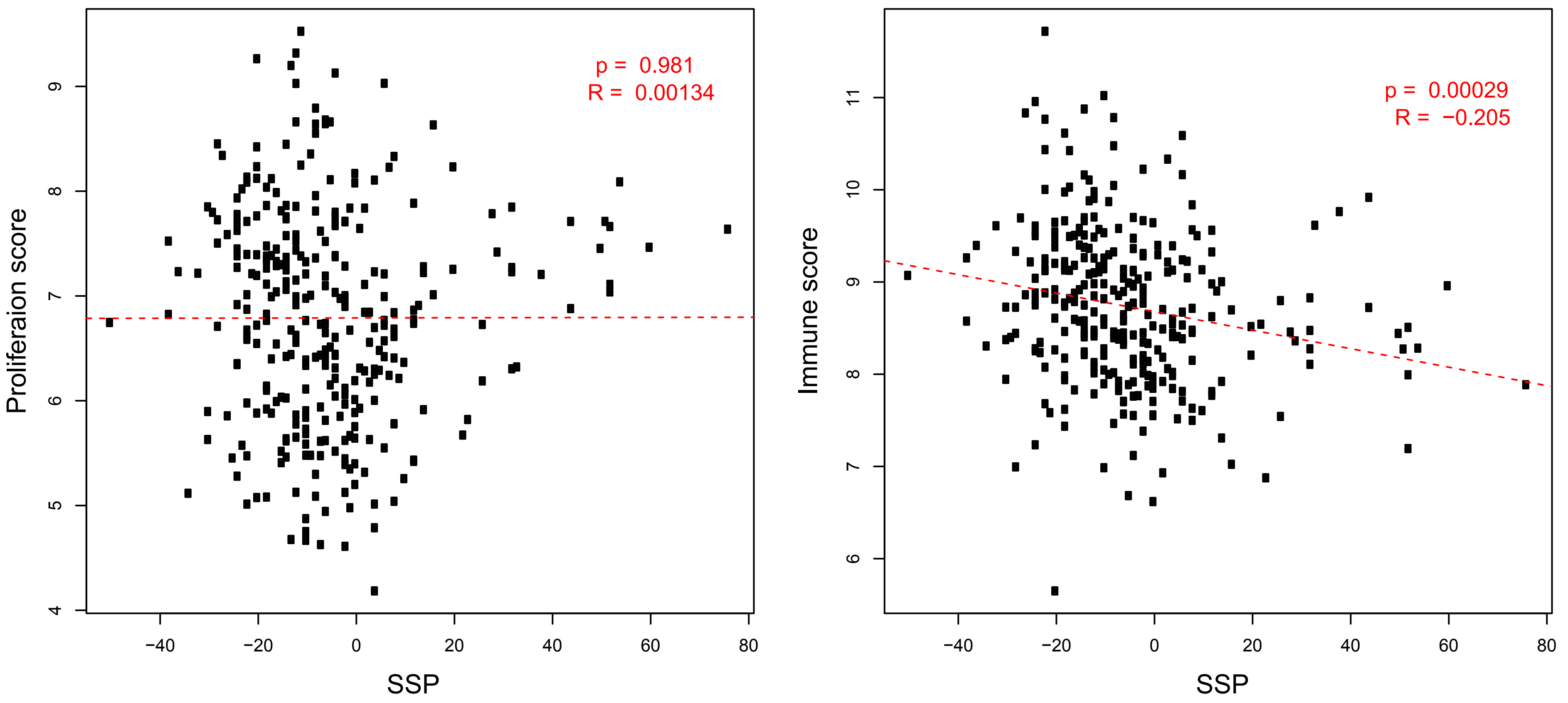

ER-RT-

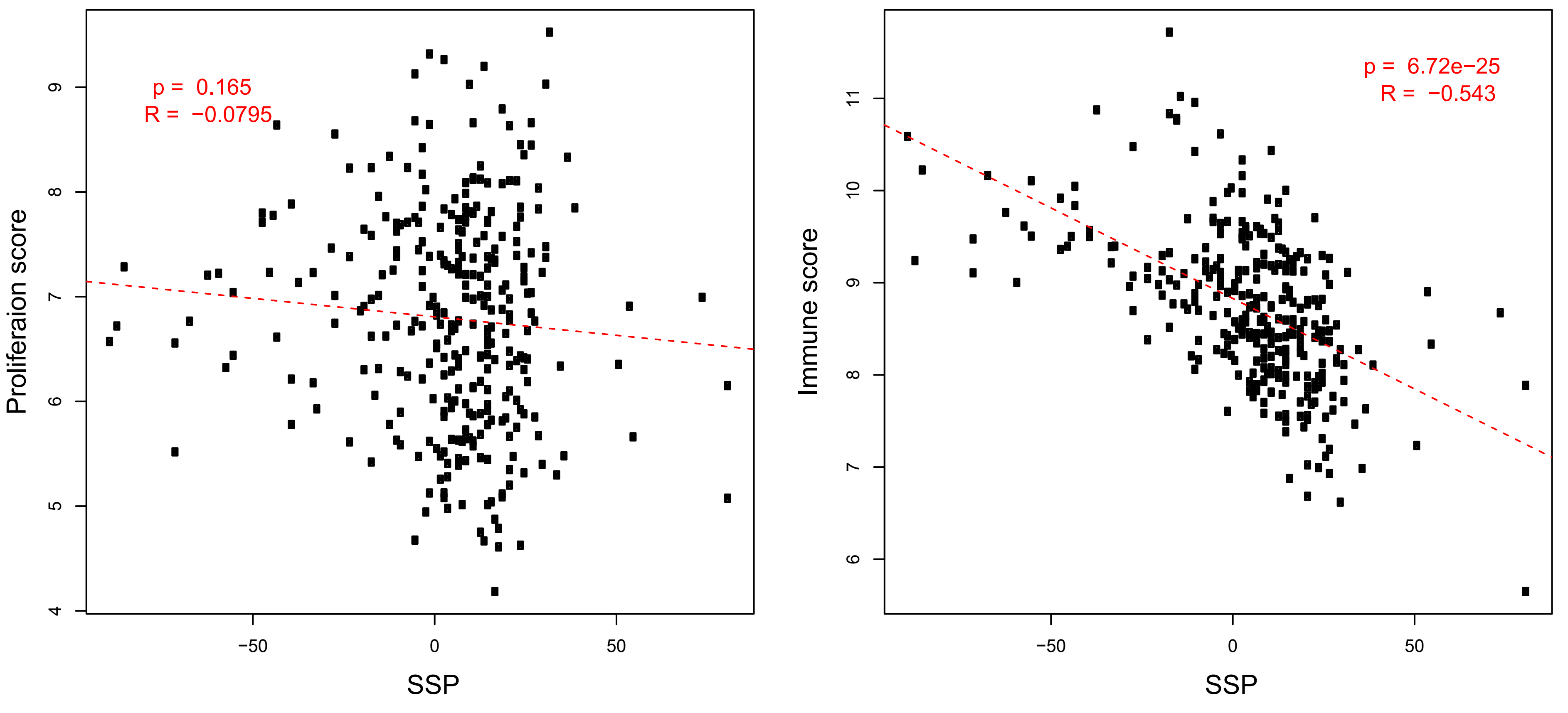

Supplement: Supplementary file 8 — Figure S4. Correlation of SSP scores with proliferation and immune response. Raw SSP scores are plotted against a proliferation score and an immune score, respectively. SSP scores are calculated based on the four different models developed stratified for estrogen receptor (ER) status and radiotherapy (RT) (ER+RT+, ER+RT-, ER-RT+, ER-RT-). Pearson correlation values and p-value from a linear model with test for zero slope are plotted together with the linear model fit. (PDF 1160 kb) [file 13058_2018_978_MOESM8_ESM.pdf]
